# Arginine methylation in cancer: mechanisms and therapeutic implications

**DOI:** 10.1186/s40364-025-00860-5

**Published:** 2025-11-07

**Authors:** Yuanyuan Xu, Qihui Wu, Yuxiu Zhang, Yijin Gu, Hui Zhu, Xiaodan Fu, Anqi Li, Yimin Li

**Affiliations:** 1 Department of Pathology, Ruijin Hospital, Shanghai Jiaotong University School of Medicine, Shanghai, 200025 China; 2 Department of Gynecology, Xiangya Hospital, Central South University, Changsha, 410008 China; 3 National Clinical Research Center for Geriatric Disorders, Xiangya Hospital, Changsha, 410008 China; 4 Department of Pathology, Zhongshan Hospital, Fudan University, Shanghai, 200032 China; 5 Department of Pathology, Shanghai Pudong New District Zhoupu Hospital, Shanghai, 200120 China; 6 Department of Pathology, Xiangya Hospital, Central South University, Changsha, 410008 China

**Keywords:** Post-translational modification, Arginine methylation, PRMTs, AdoMet, Cancer hallmarks, Targeted therapy

## Abstract

Arginine methylation is a critical post-translational modification that modulates protein stability, enzymatic activity, and subcellular localization, thereby shaping cell fate decisions and maintaining cellular homeostasis. As the principal enzymes catalyzing this modification, protein arginine methyltransferases (PRMTs) participate in key biological processes, including transcriptional and post-transcriptional regulation as well as signal transduction. Dysregulated PRMT activity has been increasingly linked to tumor initiation, progression, and therapeutic resistance. This review summarizes PRMT classification, structural and functional characteristics, and upstream regulatory mechanisms, offering a framework for understanding their diverse roles in cancer biology and therapeutic relevance. We further discuss the mechanistic contributions of PRMTs to multiple cancer hallmarks and highlight recent advances in the development of PRMT inhibitors. Finally, we examine current strategies for clinical translation, with particular emphasis on combination approaches involving chemotherapy, targeted therapy, and immunotherapy, thereby offering a foundation for advancing PRMT-targeted precision oncology.

## Introduction

Post-translational modifications (PTMs) precisely regulate protein structure and function through covalent modifications. Aberrations in PTMs can disrupt critical signaling pathways, driving tumor progression and presenting attractive targets for precision oncology [1, 2]. More than 600 distinct PTM types have been described, among which phosphorylation, acetylation, methylation, and ubiquitination play central roles in cellular signaling, epigenetic regulation, and tumorigenesis [3–5]. Arginine methylation, first identified in the 1960s, has garnered increasing attention due to its biochemical stability [6–8]. However, major advances in this field only occurred in the mid-1990s, following the cloning of protein arginine methyltransferase 1 (PRMT1) [9]. Compared to research on other PTMs, progress in arginine methylation was initially constrained by the lack of specific antibodies and effective inhibitors, which limited substrate identification and mechanistic studies. Despite recent advances in proteomics and cell biology, the complete landscape of arginine methylated substrates and their cancer-specific functions remains incompletely understood [10].

As a critical PTM, arginine methylation involves the transfer of methyl groups from S-adenosylmethionine (AdoMet, also known as SAM) to the guanidino group of arginine residues, increasing residue hydrophobicity and steric bulk while reducing hydrogen bonding capacity [11]. This modification is catalyzed by nine PRMTs identified in mammals, which regulate key processes such as gene expression and DNA damage repair [12]. Although somatic PRMT mutations are rare in cancer, aberrant overexpression is frequently observed and often correlates with poor prognosis [12]. With growing knowledge of PRMT activity, substrate specificity, and cofactor interactions, PRMTs have emerged as promising therapeutic targets in oncology [13, 14]. Encouragingly, the development of selective PRMT inhibitors has demonstrated preclinical efficacy and early clinical potential in both solid tumors and hematologic malignancies [15].

Given their diverse biological functions, PRMTs contribute to multiple cancer hallmarks, including sustained proliferation, resistance to cell death, metastasis, and DNA damage response (DDR), underscoring their central role in oncogenesis. A comprehensive understanding of these hallmark-associated functions is essential for identifying predictive biomarkers and guiding rational combination therapies. In this review, we first summarize the classification and structural characteristics of the PRMT family. We then discuss recent advances in their regulatory functions in gene expression and the upstream pathways that modulate their expression and activity. Finally, we explore their involvement in cancer hallmarks and review the progress in PRMT-targeted drug development, offering insights into their translational potential in precision oncology.

## Classification, structure, and functional features of PRMTs

### Classification of PRMTs

**Fig. 1:**
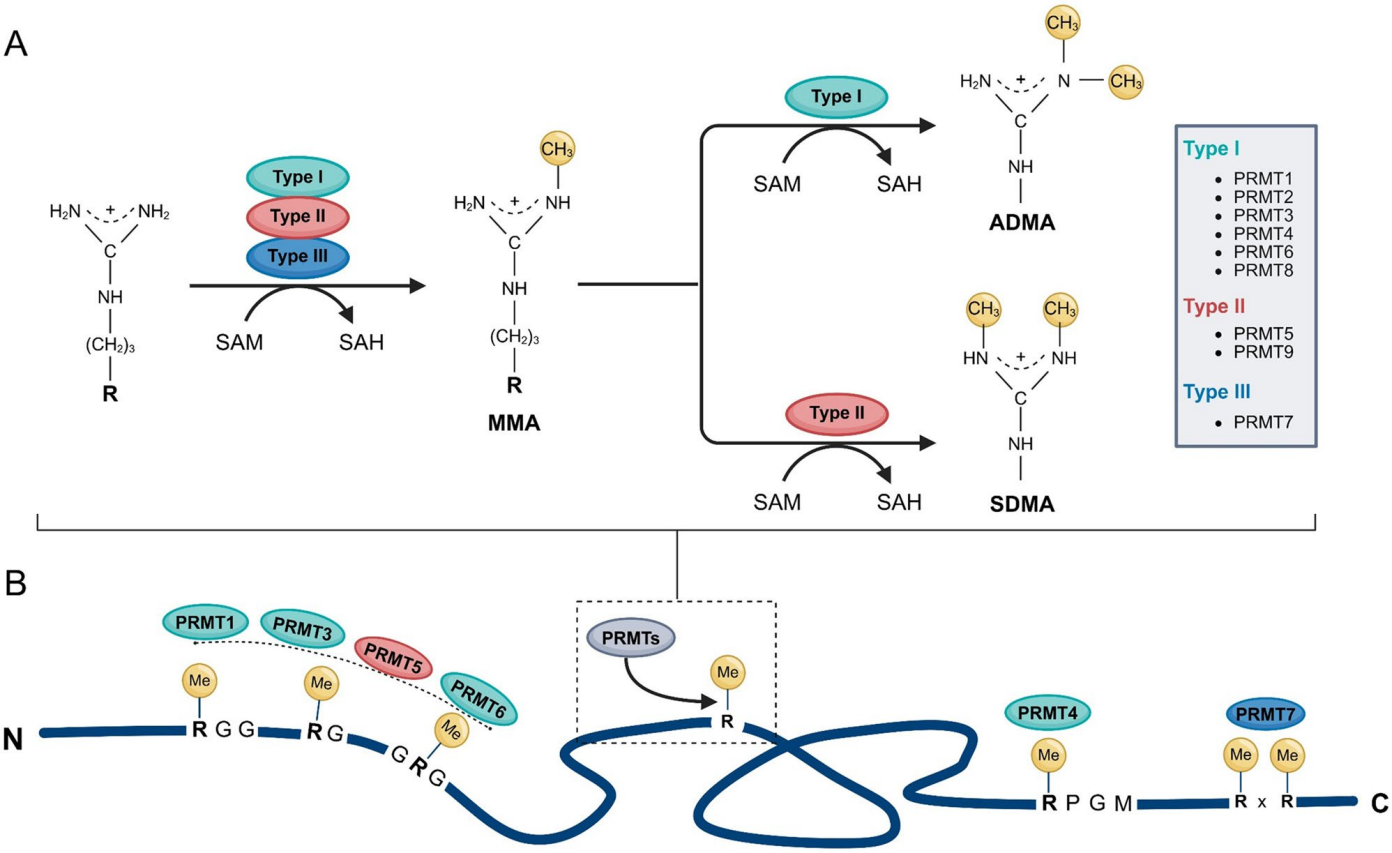
Arginine Methylation Types and Substrate Motif Recognition by PRMTs. (**A**) PRMTs catalyze the transfer of methyl groups from the methyl donor S-adenosylmethionine (AdoMet, also known as SAM) to the guanidino nitrogen atoms of arginine residues, producing S-adenosylhomocysteine (AdoHcy, also known as SAH) as a byproduct. All three PRMT types (I, II, and III) can generate ω-*N*^G^-monomethylarginine (MMA). Type I PRMTs further catalyze the formation of asymmetric ω-*N*^G^,*N*^G^-dimethylarginine(ADMA), whereas Type II PRMTs produce symmetric ω-*N*^G^,*N*^G^-dimethylarginine (SDMA). The boxed diagram on the right summarizes the classification and representative members of each PRMT type. (**B**) PRMTs exhibit substrate specificity based on arginine-containing sequence motifs. PRMT1, PRMT3, PRMT5, and PRMT6 preferentially methylate substrates containing RGG, RG, or GRG motifs. PRMT4 targets arginine residues flanked by PGM motifs, while PRMT7 primarily modifies arginine within RxR motifs. “Me” indicates arginine methylation

PRMTs are classified into three types based on the methylation pattern of the guanidino group of arginine residues. Type I PRMTs (including PRMT1-3, PRMT4/CARM1, PRMT6, and PRMT8) catalyze the monomethylation of arginine residues to generate ω-*N*^G^-monomethylarginine (MMA), followed by a second methylation on the same terminal nitrogen to form asymmetric ω-*N*^G^,*N*^G^-dimethylarginine (ADMA). Type II PRMTs (represented by PRMT5 and PRMT9) also first generate MMA, but subsequently catalyze methylation on the opposite terminal nitrogen to produce symmetric ω-*N*^G^,*N*^G^-dimethylarginine (SDMA). Type III PRMTs, represented solely by PRMT7, catalyze only monomethylation, generating MMA exclusively (Fig. 1A) [16].

### Structural basis of PRMTs

The catalytic activity of PRMTs relies on a set of highly conserved sequence motifs. Motif I (VLD/EVGxGxG), which includes three invariant glycine residues, forms the core of the AdoMet binding pocket. The adjacent Post-I motif (V/IxG/AxD/E), enriched in acidic residues, stabilizes AdoMet via hydrogen bonding with its ribose hydroxyl group. Motifs II (F/I/VDI/L/K) and III (LR/KxxG) cooperate to form a β-sheet structure that maintains the integrity of the AdoMet binding site. Additionally, the conserved THW loop adjacent to the catalytic site contributes to substrate recognition and positioning [17–19]. Collectively, these motifs orchestrate AdoMet binding, proton transfer, and substrate recognition, thereby ensuring efficient arginine methylation.

Beyond these conserved catalytic elements, several PRMTs possess non-catalytic domains that expand their functional repertoire. For example, PRMT2 harbors an N-terminal Src homology 3 (SH3) domain—approximately 50 amino acids forming a five-stranded β-barrel—that facilitates protein-protein interactions by recognizing proline-rich motifs [20]. PRMT3 contains a zinc finger domain at its N-terminus, which interacts with RNA, proteins, and lipids in addition to DNA [21]. PRMT4 features a C-terminal transcriptional activation domain (TAD) that interacts with transcriptional co-regulators to modulate gene expression [13]. PRMT5 includes an N-terminal TIM-barrel domain that promotes dimerization, enhances substrate binding, and mediates interaction with methylosome protein 50 (MEP50, also known as p44 or WDR77), forming a hetero-octameric PRMT5-MEP50 complex with greater enzymatic activity than PRMT5 homodimers [22, 23]. As the only known type III PRMT, PRMT7 contains tandem methyltransferase domains at both termini, forming a pseudo-dimer that enables catalytic activity independent of conventional dimerization. PRMT8 possesses a unique N-terminal myristoylation signal that mediates membrane localization [24]. PRMT9 is structurally distinct, characterized by an extended core enriched in β-folds and an N-terminal region containing tetratricopeptide repeat motifs, putatively serving as a scaffold for protein–protein interactions [25, 26].

### PRMT catalytic mechanism and substrate specificity

Most PRMTs function as catalytically active homodimers, with subunits arranged in a head-to-tail configuration. However, PRMT7 and PRMT9, which likely arose from gene duplication events, each contain two tandem methyltransferase domains that fold into a pseudo-dimeric structure, enabling enzymatic activity without requiring dimerization. The PRMT catalytic cycle consists of several sequential steps: (1) the methyl donor AdoMet binds to the Rossmann fold within the methyltransferase domain, stabilized by hydrophobic interactions and hydrogen bonds involving Motif I and the Post-I motif; (2) the guanidino group of the substrate arginine is positioned at the active site through interactions with acidic residues in the C-terminal β-barrel-like domain and π–cation stacking with the conserved tryptophan in the THW loop; (3) the methyl group is transferred from AdoMet to the substrate, generating MMA; (4) the methylated product and the byproduct S-adenosylhomocysteine (AdoHcy, also known as SAH) dissociate, allowing the enzyme to reset for a new catalytic cycle; and (5) in Type I and Type II PRMTs, a subsequent catalytic round introduces a second methyl group, yielding ADMA or SDMA, respectively [11, 14, 15, 18].

Despite the conserved catalytic core, PRMTs exhibit remarkable substrate specificity. This diversity arises from variations in their auxiliary domains (e.g., SH3, TAD, TIM-barrel) and from sequence and structural features of their protein substrates. Specifically, the amino acid composition, charge distribution, local conformation, and hydrophobicity surrounding the target arginine residue strongly influence selectivity. For example, PRMT1, PRMT3, PRMT5, and PRMT6 preferentially methylate glycine–arginine-rich (GAR) motifs, whereas PRMT4 favors arginine adjacent to proline–glycine–methionine (PGM) motifs (Fig. 1B). PRMT7 specifically recognizes arginines within an RxR motif, where two arginines are separated by a single intervening residue, commonly found in RNA-binding proteins and nuclear regulators (Fig. 1B) [12, 27–29]. These recognition mechanisms underlie the functional diversity of PRMTs in processes such as chromatin remodeling, RNA metabolism, and signal transduction, while also providing a structural framework for the development of selective inhibitors.

## Biological functions of PRMTs

Arginine methylation is a prevalent PTM that plays a critical role in regulating transcription, diverse post-transcriptional events (such as pre-mRNA splicing, mRNA stability, and translation), and multiple signal transduction pathways. Beyond their enzymatic activity, PRMTs also exert regulatory functions through non-catalytic mechanisms such as scaffolding interactions, thereby broadening their influence on gene expression and cellular physiology. Collectively, these activities underscore the multifaceted regulatory roles of PRMT-mediated arginine methylation in cancer biology and highlight its potential therapeutic relevance.

### The role of PRMTs in transcriptional regulation

**Fig. 2:**
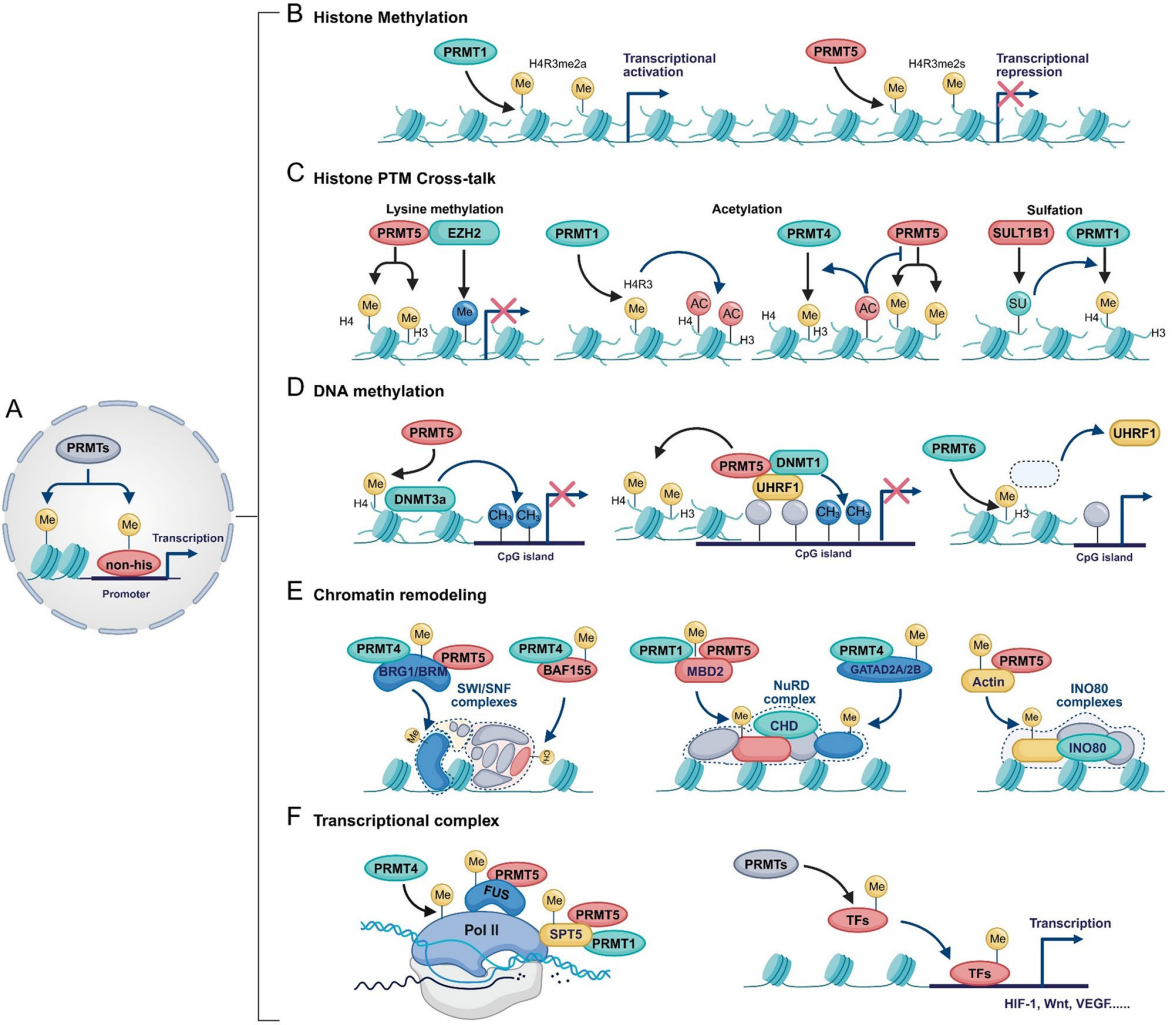
Mechanisms of PRMTs in Transcriptional Regulation. (**A**) Schematic overview of PRMT-mediated transcriptional regulation through histone and non-histone arginine methylation. Yellow “Me” indicates arginine methylation. (**B**) PRMTs modulate gene transcription by catalyzing histone arginine methylation. For instance, PRMT1-mediated H4R3me2a is typically associated with transcriptional activation, while PRMT5-mediated H4R3me2s is linked to transcriptional repression. (**C**) Crosstalk between histone arginine methylation and other post-translational modifications (PTMs). Left: PRMT5 cooperates with EZH2 to catalyze both arginine and lysine methylation, synergistically repressing transcription. Middle: H4R3me2a promotes histone acetylation, and conversely, acetylation can influence PRMT-mediated methylation. Right: Histone tyrosine sulfation by SULT1B1 enhances PRMT1-catalyzed H4R3me2a. Blue “Me” indicates lysine methylation; “AC” indicates acetylation; “SU” indicates tyrosine sulfation. (**D**) PRMTs indirectly regulate DNA methylation. Left: PRMT5-mediated H4R3me2s facilitates the recruitment of DNMT3A. Middle: PRMT5 and DNMT1 act cooperatively to modulate both histone and DNA methylation. Right: PRMT6-catalyzed H3R2me2a disrupts UHRF1 binding to H3, leading to passive DNA demethylation. (**E**) PRMTs contribute to chromatin remodeling by methylating components of chromatin remodeling complexes, including SWI/SNF (left), NuRD (middle), and the INO80 complex (right). (**F**) PRMTs regulate transcriptional activity by methylating core components of the transcriptional machinery, including RNA polymerase II (Pol II) and its cofactors such as FUS and SPT5 (left), as well as specific transcription factors (TFs) (right)

**Fig. 3:**
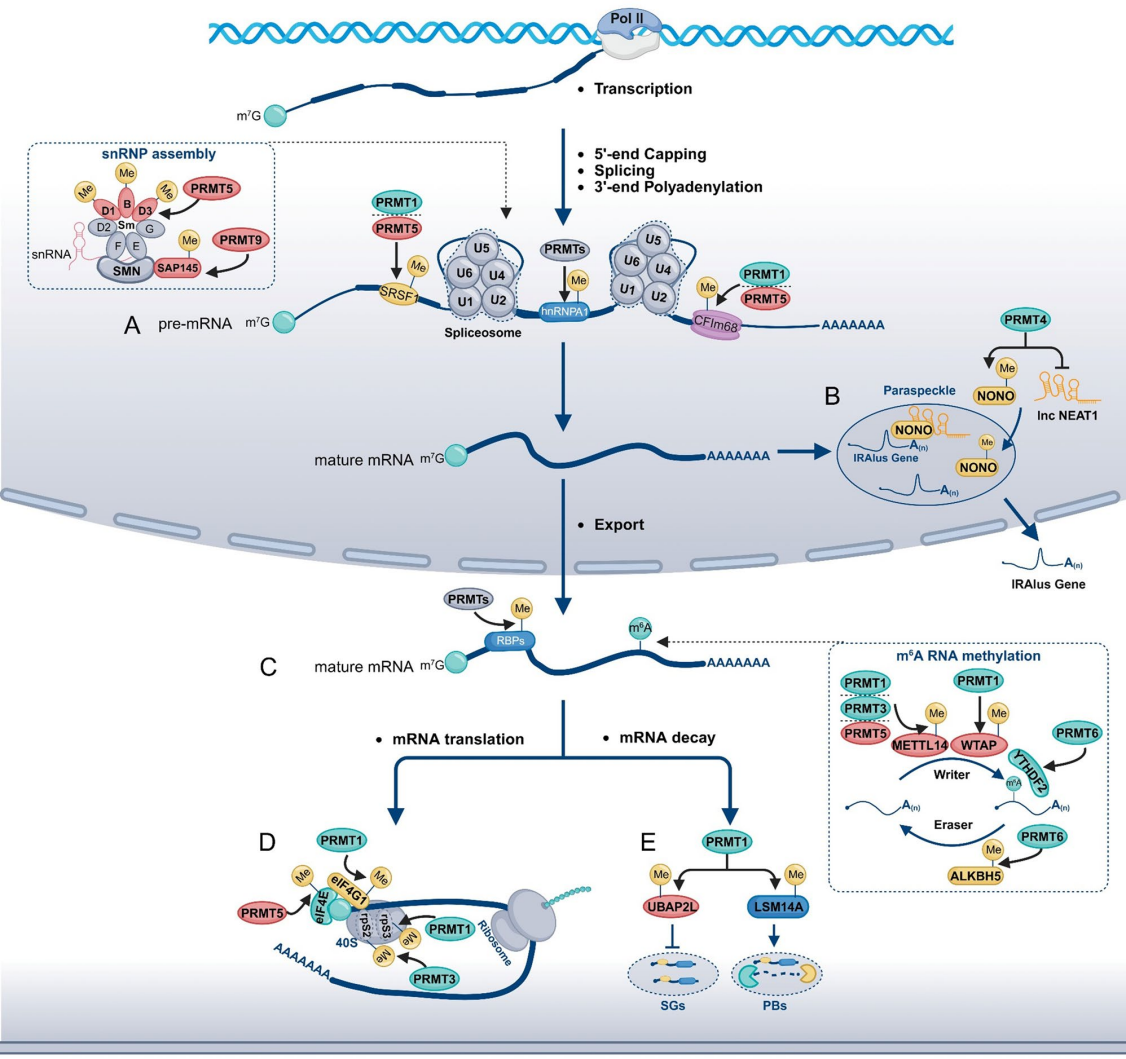
Roles of PRMTs in Post-Transcriptional Regulation of Gene Expression. (**A**) PRMTs regulate pre-mRNA splicing by methylating small nuclear ribonucleoprotein (snRNP) subunits and splicing factors such as SRSF1, hnRNPA1, and CFIm68. (**B**) PRMTs modulate the formation of paraspeckle, influencing the nuclear retention of mRNAs containing inverted repeat Alu elements (IRAlus). (**C**) PRMTs affect mRNA stability by methylating RNA-binding proteins (RBPs) or factors associated with m^6^A RNA methylation. (**D**) PRMTs regulate mRNA translation by modifying components of the eIF4F initiation complex and the 40 S ribosomal subunit. (**E**) PRMTs influence mRNA decay by modulating the assembly of stress granules (SGs) and processing bodies (PBs)

**Fig. 4:**
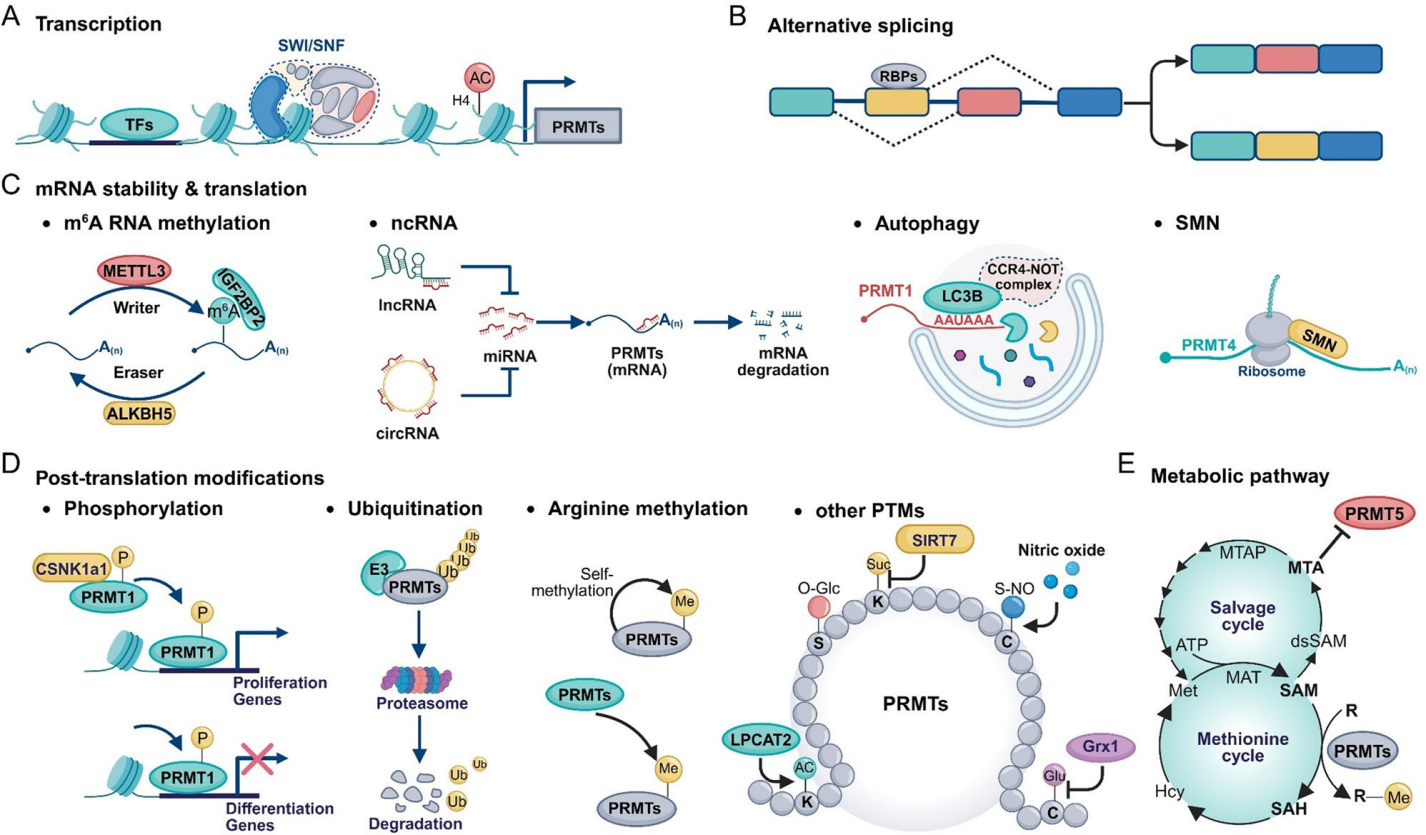
Upstream Regulatory Network of PRMTs. (**A**) PRMT transcription is regulated by diverse upstream factors, including TFs, SWI/SNF complexes, and histone acetylation modifications. (**B**) RBPs modulate alternative splicing of PRMT pre-mRNAs, contributing to isoform diversity and functional heterogeneity. (**C**) PRMT mRNA stability and translation are influenced by multiple mechanisms, such as m⁶A RNA modification (left), ncRNAs (middle left), autophagy (middle right), and the SMN protein (right). (**D**) PRMTs undergo various PTMs that regulate enzymatic activity and protein stability. Examples include: CSNK1α1-mediated phosphorylation of PRMT1, which promotes transcription of proliferation-related genes while repressing differentiation-associated genes (left); ubiquitination by E3 ligases leading to PRMT degradation (middle left); automethylation or methylation by other PRMTs (middle right); and additional modifications such as acetylation (AC), O-GlcNAcylation (O-Glc), succinylation (Suc), S-nitrosylation (S-NO), and glutathionylation (Glu) (right). (**E**) Metabolism regulates PRMT enzymatic activity by modulating intracellular levels of AdoMet (SAM), AdoHcy (SAH), and methylthioadenosine (MTA). AdoMet, synthesized from methionine and ATP via methionine adenosyltransferase (MAT), serves as the methyl donor for PRMT-catalyzed methylation, generating AdoHcy. In the polyamine biosynthesis pathway, AdoMet is also converted to MTA, which can be recycled to methionine via methylthioadenosine phosphorylase (MTAP)

PRMTs regulate gene transcription through several mechanisms (Fig. 2A): (1) direct methylation of histone arginine residues, which modulates chromatin structure and DNA accessibility (Fig. 2B); (2) cross-talk with other histone modifications—such as lysine methylation, acetylation, or tyrosine sulfation—resulting in either synergistic or antagonistic effects on gene expression (Fig. 2C); (3) indirect regulation of DNA methylation, thereby linking histone and DNA modification networks (Fig. 2D); (4) methylation of subunits within chromatin remodeling complexes, influencing chromatin conformation and transcriptional complex assembly (Fig. 2E); and (5) methylation of other non-histone proteins, such as RNA polymerase II (Pol II) and various transcription factors, thereby modulating their activity or interactions (Fig. 2F). Collectively, these mechanisms underscore the central role of PRMTs as epigenetic regulators that integrate histone and non-histone methylation to fine-tune transcriptional programs.

#### PRMTs-mediated methylation of histones

Histones H2A, H2B, H3, and H4 assemble into octamers, around which approximately 147 base pairs of DNA are wrapped to form nucleosomes—the fundamental repeating units of chromatin [30, 31]. The N-terminal tails of histones harbor abundant modifiable residues that undergo reversible PTMs, which regulate chromatin structure and function either by altering the physical properties of chromatin or by modulating the recruitment of effector proteins [2]. Among these PTMs, arginine methylation plays a pivotal role in chromatin remodeling, transcriptional control, and the DNA damage response [32]. Distinct PRMTs catalyze methylation at specific histone arginine residues, conferring precise and context-dependent regulatory effects [33, 34].

##### Type I PRMTs

PRMT1 primarily catalyzes the asymmetric dimethylation of histone H4R3 (H4R3me2a), a modification commonly associated with gene transcription activation (Fig. 2B) [35, 36]. Beyond H4R3, PRMT1 also targets H2A residues (R3, R11, R29) [37]. Although PRMT2 has been reported to catalyze the H3R8me2a, its catalytic efficiency is lower and may depend on other cofactors or protein interactions [38, 39]. PRMT3 and PRMT8 can methylate histone H4 in its free form under in vitro [40, 41]. PRMT4 mainly catalyzes the methylation of H3 at R17, R26, and R42, and facilitates transcriptional activation by recruiting transcriptional co-activators such as p300/CBP and switch/sucrose non-fermentable (SWI/SNF) complexes, promoting chromatin accessibility. PRMT6-catalyzed H3R2me2a is enriched in promoter regions, where it forms an antagonistic relationship with H3K4me2/3, hindering the binding of transcription initiation complexes, thereby inhibiting gene expression [42]. PRMT6 can also methylate H4R3 and multiple H2A sites (R3, R11, R29), with H2AR29me2 frequently linked to repression [37]. Notably, PRMT6 exhibits automethylation activity, which may modulate its own function [27, 43].

##### Type II PRMTs

Unlike PRMT1, PRMT5 predominantly catalyzes symmetric dimethylation of H4R3 (H4R3me2s), which serves as a repressive mark by recruiting complexes such as DNMT3A and HDACs, leading to chromatin condensation and transcriptional silencing (Fig. 2B) [44]. PRMT5 also targets H2AR3 and H3R8, reinforcing repression [45–47]. Interestingly, PRMT5 also catalyzes H3R2me1 and H3R2me2s, which promote H3K4 trimethylation (H3K4me3) by recruiting WD repeat domain 5 (WDR5), thereby enhancing Pol II recruitment and transcriptional activation [48–51].

##### Type III PRMTs

PRMT7 primarily catalyzes the monomethylation of histones arginine residues (e.g., H2AR3me1, H4R3me1, H3R2me1), collaborating or competing with other PRMTs to establish a repressive chromatin environment [52, 53]. Additionally, PRMT7 can methylate arginine residues in the RxR motif of histones (e.g., H4R17/19, H2BR29/31/33), though its precise functions remain to be further investigated [54, 55].

#### Cross-talk between arginine methylation and other histone modifications

Histone PTMs are essential for regulating gene expression, DNA repair, replication, and chromatin architecture [56]. Growing evidence suggests that these modifications act through mutual interactions or synergistic mechanisms [57]. In particular, recent studies have revealed extensive crosstalk between arginine methylation and other histone marks, including lysine methylation, acetylation, and tyrosine sulfation. Rather than functioning independently, these PTMs operate in a coordinated manner to shape a dynamic epigenetic regulatory network [58].

##### Histone lysine methylation

The Polycomb repressive complex 2 (PRC2) catalyzes H3K27 trimethylation (H3K27me3), contributing to chromatin compaction and transcriptional repression [59]. EZH2, the catalytic subunit of PRC2, is methylated by PRMT1 at R342, which protects it from ubiquitin-mediated degradation [60]. In colorectal cancer (CRC), PRMT5-deposited H4R3me2s and H3R8me2s co-localize with EZH2-mediated H3K27me3 at the CDKN2B promoter, synergistically repressing transcription (Fig. 2C) [61]. Interestingly, in acute myeloid leukemia (AML), loss of PRMT5 leads to a global increase in H3K27me3 levels. Mechanistically, although PRMT5 does not directly regulate PRC2 enzymatic activity, its methylation of histone H3 interferes with subsequent PRC2-mediated trimethylation, highlighting a complex and tumor-specific interplay between arginine and lysine methylation.

##### Histone acetylation

Histone lysine acetylation occurs at over 40 residues, playing diverse roles in chromatin regulation [62]. PRMT1-mediated H4R3 methylation promotes histone acetylation, whereas PRMT1 depletion reduces global acetylation levels (Fig. 2C) [63–66]. Conversely, acetylation can modulate PRMT activity and methylation type, thus influencing chromatin state and gene expression. For example, H3K9ac and H3K14ac inhibit PRMT5-mediated symmetric dimethylation of H3R8 and H4R3 [67], while sequential acetylation at H3K18 and H3K23 enhances PRMT4 recruitment to chromatin and facilitates asymmetric dimethylation of H3R17 (Fig. 2C) [68]. Additionally, H4K5ac suppresses PRMT1-catalyzed H4R3 methylation but promotes PRMT5-mediated modification at the same site, switching the mark from transcriptionally activating ADMA to repressive SDMA [69].

##### Histone tyrosine sulfation

Tyrosine sulfation involves the addition of a sulfate group to the phenolic side chain of tyrosine residues, producing sulfotyrosine [70]. This modification is common on extracellular and membrane proteins, where it regulates functions such as cell adhesion, blood coagulation, inflammation, and pathogen infection [70]. Recent evidence shows that newly synthesized histone H3 in the cytoplasm can be sulfated at Y99 by the sulfotransferase SULT1B1, which enhances PRMT1 binding to chromatin and regulates H4R3me2a levels and gene transcription (Fig. 2C) [71].

#### PRMTs indirectly regulate DNA methylation

DNA methylation is a key epigenetic modification involved in transcriptional regulation, organismal growth and development, as well as cancer initiation and progression [72–74]. This modification is catalyzed by DNA methyltransferases, including the maintenance enzyme DNMT1 and the de novo enzyme DNMT3A and DNMT3B [75]. Previous studies have demonstrated that PRMT5-mediated H4R3me2s recruits DNMT3A, thereby linking histone methylation to DNA methylation during gene silencing (Fig. 2D) [76, 77]. UHRF1, a key DNMT1 cofactor often overexpressed in cancers, binds hemimethylated CpG sites and recruits DNMT1 to maintain DNA methylation [78, 79]. PRMT5 can interact with UHRF1 to co-regulate histone and DNA methylation (Fig. 2D) [80, 81]. Notably, UHRF1 binds preferentially to unmethylated H3R2, and methylation at this site inhibits the interaction [82]. Overexpression of PRMT6 elevates H3R2me2a, thereby impairing UHRF1 binding to histone H3 and resulting in passive DNA demethylation (Fig. 2D) [83, 84]. Collectively, these findings indicate that PRMTs can modulate DNA methylation indirectly, shaping the epigenetic landscape and influencing gene expression regulation.

#### PRMTs and chromatin remodeling

The human genome is compacted into chromatin, a dynamic structure composed of DNA, histones, and other associated proteins. Chromatin transitions between open euchromatin and condensed heterochromatin states regulate DNA accessibility and transcription [85]. This dynamic remodeling is orchestrated by histone modifications and ATP-dependent chromatin remodeling complexes, which utilize ATP hydrolysis to reposition, eject, or restructure nucleosomes and thereby control gene expression [86]. All ATP-dependent remodeling complexes contain catalytic ATPase/helicase subunits of the SWI2/SNF2 family and are classified into four subfamilies based on ATPase homology and associated cofactors: SWI/SNF, ISWI, chromodomain helicase DNA-binding (CHD), and inositol 80 (INO80) [87]. Specialized complexes like the human Tat-interactive protein, 60 kDa (TIP60) complex also contribute to chromatin remodeling. Beyond histone methylation, PRMTs modulate chromatin structure by methylating core or accessory subunits of these complexes (Fig. 2E).

##### SWI/SNF complex

The SWI/SNF complex comprises catalytic ATPase subunits (e.g., Brahma (BRM) or BRM-related gene 1 (BRG1)), core components (e.g., BRM-associated factor 155 (BAF155), BAF170, BAF47), and variable auxiliary subunits (e.g., BAF57, BAF53A/B, BAF60A/B/C, β-actin) [87, 88]. PRMT4 and PRMT5 physically interact with BRG1 and BRM, modulating chromatin accessibility and transcription (Fig. 2E) [67, 89–91]. PRMT4 also methylates BAF155 (Fig. 2E), enhancing its recruitment to c-Myc target sites and facilitating the switch from SWI/SNF to the EZH2 complex, leading to repression of MAD2L2 [92, 93]. These findings highlight the intricate interplay between PRMTs and the SWI/SNF complex in epigenetic regulation.

##### CHD family and NuRD complex

The CHD family comprises conserved ATP-dependent chromatin remodelers that play essential roles in development and gene regulation [94]. Among them, CHD3 and CHD4 serve as key catalytic subunits of the nucleosome remodeling and deacetylase (NuRD) complex, which is involved in transcriptional repression, DNA repair, and lineage commitment [95, 96]. Although the direct mechanistic relationship between PRMTs and CHD3/4 remains to be fully elucidated, PRMTs regulate NuRD function by methylating accessory subunits such as MBD2 and GATAD2A/B [97, 98]. Specifically, PRMT1 and PRMT5 methylate MBD2, reducing its DNA-binding affinity and repressive activity (Fig. 2E) [97]. Moreover, PRMT4 catalyzes methylation of arginine-rich domains within GATAD2A/B, thereby facilitating NuRD complex assembly and promoting the G1/S cell cycle transition through transcriptional activation of cell cycle–related genes (Fig. 2E) [98].

##### INO80 complex

The INO80 complex contains a catalytic subunit, INO80, and regulatory subunits including actin and actin-related proteins (Arp4, Arp5, Arp8) [99]. Among them, actin not only serves as a scaffold to mediate the complex’s positioning at specific genomic regions, but also generates mechanical forces through polymerization/depolymerization, promoting nucleosome remodeling [100]. PRMT5 monomethylates actin at R256, modulating its interaction with Pol II/III and the INO80 complex, thereby influencing transcriptional activity and disease-associated gene regulation (Fig. 2E) [101].

##### TIP60 complex

The TIP60 complex, derived from yeast NuA4 and SWR1, regulates chromatin accessibility via histone acetylation and interactions with INO80-family remodelers [102–105]. PRMT4 and PRMT5 modulate TIP60 activity by methylating its coactivators, including RuvB-like protein 1 (RUVBL1) and Pontin [106, 107]. Specifically, PRMT5-mediated methylation of RUVBL1 at arginine 205 enhances TIP60’s acetyltransferase activity, promotes H4K16 acetylation, and facilitates homologous recombination (HR) by displacing p53-binding protein 1 (53BP1) from DNA damage sites [106]. Under glucose starvation, PRMT4 methylates Pontin, promoting TIP60 recruitment and H4K16 acetylation, which in turn activates FOXO3a-dependent transcription of autophagy-related genes [107].

#### PRMT-mediated methylation of other non-histone proteins

Although the majority of studies have focused on histone modification, increasing evidence indicates that PRMTs also regulate the methylation of non-histone proteins, which play critical roles in transcription. Beyond chromatin remodeling complexes, PRMTs target proteins such as Pol II, transcription elongation factors, and diverse transcription factors, thereby highlighting their multifaceted roles in orchestrating gene expression and maintaining cellular homeostasis.

Transcriptional elongation by Pol II is a dynamic process essential for implementing gene expression programs, and PTMs of its carboxy-terminal domain (CTD) are crucial for initiation and elongation [108, 109]. PRMT4 methylates Pol II at R1810, modulating initiation and promoting the expression of small nuclear and small nucleolar RNAs (Fig. 2F). Fused in sarcoma (FUS) forms phase-separated droplets that mediate its interaction with the Pol II CTD [110–112], and PRMT5-catalyzed symmetric dimethylation of FUS is required for its stable association with Pol II (Fig. 2F) [113]. Additionally, PRMT1 and PRMT5 methylate the transcription elongation factor SPT5 at R681, R696, and R698, modulating its interaction with Pol II and contributing to transcriptional elongation (Fig. 2F) [114]. PRMTs also directly modulate transcription factors by catalyzing selective asymmetric or symmetric methylation on critical arginine residues, thereby fine-tuning DNA-binding affinity, protein stability, and co-regulator recruitment (Fig. 2F). Notable targets include p53, c-Fos, NF-κB (p65/RelA), RUNX1, C/EBPα, Twist1, PGC-1α, Gli1, and c-Myc [115–124]. These factors are central regulators of cell cycle progression and oncogenic pathways, including HIF-1, Wnt, and VEGF signaling, with dysregulation closely linked to tumor initiation and progression.

### PRMT-mediated regulation of the mRNA lifecycle

The mRNA lifecycle—from transcription to degradation—encompasses a series of tightly regulated processes, including splicing, nuclear export, localization, translation, and decay. These events are orchestrated by an intricate network of regulators, such as the small nuclear ribonucleoproteins (snRNPs) assembly, RNA-binding proteins (RBPs), N(6)-methyladenosine (m^6^A) RNA methylation, and translation initiation complexes, ensuring gene expression is both spatially and temporally controlled [125–129]. PRMTs have emerged as pivotal modulators across multiple stages of this lifecycle, influencing splicing, nuclear export, mRNA stability, and translational efficiency.

#### PRMTs influence Pre-mRNA splicing and 3’ end processing

##### SnRNP assembly

Pre-mRNA splicing requires the accurate assembly and dynamic remodeling of the spliceosome, as well as precise activation of its catalytic core. Spliceosomes are classified into U2-type (major) and U12-type (minor) complexes, with U2-type accounting for most splicing events [130, 131]. Efficient spliceosome function relies on the proper assembly and maturation of snRNPs, a process tightly regulated by PTMs, many of which are catalyzed by PRMTs [132–134]. PRMT5, in complex with cofactors such as pICln and MEP50, catalyzes symmetric dimethylation of Sm proteins (e.g., SmB/B’, D1, D3), promoting their transfer to snRNAs via the survival motor neuron (SMN) complex and thereby facilitating snRNP assembly and spliceosome maturation (Fig. 3A) [132, 133]. Loss of PRMT5 disrupts proper snRNP biogenesis and leads to widespread splicing dysregulation across diverse cell types [135–139]. In addition, PRMT9 methylates the splicing factor SAP145 at R508, generating a binding site for the Tudor domain of SMN, further supporting snRNP biogenesis (Fig. 3A) [134].

##### Trans-acting factors

Splicing is also regulated at the level of splice site selection, coordinated by cis-acting sequence elements and trans-acting factors. Among the latter, Serine/arginine-rich splicing factors (SRSFs) and heterogeneous nuclear ribonucleoproteins (hnRNPs) bind pre-mRNA and modulate exon inclusion or exclusion [140–143]. PRMTs regulate splicing by methylating these trans-acting factors, thereby influencing their subcellular localization, RNA-binding capacity, and splicing activity [144–148]. For example, PRMT1 and PRMT5 methylate SRSF1 at R93, R97, and R109, modulating nuclear localization, pre-mRNA binding, and splice site recognition (Fig. 3A) [149–152]. Similarly, hnRNPA1 is methylated by PRMT3, PRMT4, PRMT5, and PRMT7, with methylation affecting alternative splicing patterns relevant to tumor progression and therapy resistance (Fig. 3A) [153, 154].

##### 3’ end processing

The generation of mature mRNA 3’ ends is a critical step in transcript maturation [155]. The cleavage factor Im (CFIm) complex—composed of CFIm25 (NUDT21), CFIm68 (CPSF6), and CFIm59 (CPSF7)—regulates pre-mRNA 3’ end processing and alternative polyadenylation [156, 157]. PRMT7 maintains mRNA homeostasis by monomethylating CFIm25 at R15, thereby influencing 3’ polyadenylation site selection, which affects 3’ UTR length, mRNA stability, and translational efficiency [158]. Concurrently, PRMT5/MEP50 symmetrically dimethylates CFIm68, while PRMT1 catalyzes its asymmetric dimethylation, with both modifications modulating mRNA 3’ end processing and splicing (Fig. 3A) [159].

#### PRMTs regulate nuclear retention

RNA nuclear retention refers to the phenomenon in which certain RNA molecules are retained within the nucleus after transcription, often due to incomplete processing (e.g., defective splicing, absence of a poly(A) tail), lack of appropriate nuclear export signals, or interaction with nuclear structures like paraspeckles. This retention leads to nuclear RNA accumulation and modulates gene expression [160–164].

Alu elements, primate-specific short interspersed nuclear elements (~ 300 nt), are present in over one million copies, comprising approximately 11% of the human genome [165]. Closely positioned, oppositely oriented Alu transcripts can form double-stranded RNA structures termed inverted Alu repeats (IRAlus) [166]. Paraspeckles selectively bind mRNAs containing IRAlus, preventing their export to the cytoplasm and thereby regulating mRNA availability and translation [165, 167]. PRMT4 regulates nuclear retention of IRAlus-containing mRNAs via two coordinated mechanisms: (1) methylation of the non-POU domain-containing octamer-binding (NONO, also known as p54^nrb^) protein, reducing its binding affinity for these transcripts; and (2) suppression of NEAT1 transcription, a long noncoding RNA essential for paraspeckle formation (Fig. 3B) [168]. Together, these effects facilitate the nuclear export of IRAlus-containing mRNAs.

#### PRMTs affect mRNA stability

##### RBPs

PRMTs regulate mRNA stability by catalyzing arginine methylation of RBPs, thereby dynamically modulating their interactions with target transcripts (Fig. 3C) [158, 169, 170]. For example, PRMT1 asymmetrically dimethylates HSP70 at R416 and R447, enhancing its binding to AU-rich elements (ARE) in BCL2 mRNA’s 3’ UTR, thereby stabilizing the mRNA and upregulating BCL2 protein expression [169]. Similarly, PRMT4 methylates HuR (ELAVL1) at R217, promoting its binding to ARE-containing mRNAs and thereby enhancing their stability and expression [170, 171].

##### m^6^A RNA methylation

Beyond direct regulation of RBPs, PRMTs regulate mRNA stability by modulating the methylation of key proteins involved in m^6^A modification, including METTL14, WTAP, ALKBH5, and YTHDF2 (Fig. 3C) [172–177]. METTL14, a core component of the methyltransferase complex catalyzing m⁶A RNA methylation, is methylated by multiple PRMTs with distinct effects [178]. PRMT1 enhances METTL14 RNA-binding and catalytic activity, increasing global m⁶A levels [172]; PRMT3 methylation reduces METTL14 stability [173]; and PRMT5 promotes METTL3-METTL14 complex activity, upregulating m⁶A-modified gene expression (Fig. 3C) [174]. Additionally, PRMT1 methylates WTAP, another component of the m^6^A methyltransferase complex, boosting m^6^A modification and stabilizing NDUFS6 mRNA [176]. PRMT6 methylates the RNA demethylase ALKBH5, increasing its enzymatic activity, which stabilizes LDHA mRNA and promotes aerobic glycolysis [175]. Beyond direct methylation, PRMT6 cooperates with CDK9 to transcriptionally activate YTHDF2 expression, facilitating degradation of APC and GSK3β mRNAs [177].

#### PRMTs regulate mRNA translation and decay

##### mRNA translation

The eIF4F complex, composed of eIF4A, eIF4E, and eIF4G, is essential for cap-dependent translation initiation in eukaryotes [179]. PRMT1 promotes eIF4F complex assembly and global translation by methylating eIF4G1, thereby driving tumor initiation and progression (Fig. 3D) [180]. Similarly, PRMT5 enhances eIF4E-dependent cap recognition, facilitating the translation of oncogenic proteins such as c-Myc, Cyclin D1, and HIF-1α (Fig. 3D) [181]. Beyond initiation, PRMTs also regulate ribosomal proteins: PRMT1-mediated methylation of RPS3 maintains its nucleolar localization and proper ribosome assembly, whereas PRMT3-dependent asymmetric dimethylation of RPS2 is required for ribosome biogenesis and translational activity (Fig. 3D) [182, 183].

##### mRNA decay

Stress granules (SGs) and processing bodies (PBs) are membrane-less messenger ribonucleoprotein (mRNP) granules in the cytoplasm that temporarily sequester translationally repressed mRNAs, thereby regulating gene expression under stress conditions [184, 185]. UBAP2L, a conserved factor involved in proteostasis and aggregation, is essential for SG formation [186, 187]. PRMT1-mediated methylation of its RGG motif inhibits UBAP2L’s interaction with SG components, thereby preventing SG assembly (Fig. 3E) [188]. P-bodies are enriched in proteins involved in translational repression and mRNA decay, acting as key regulators of mRNA fate [189]. LSM14A (also known as RAP55A), a translation repressor localized to both SGs and PBs, regulates their functions by interacting with mRNP complexes [190]. PRMT1 methylates multiple arginine residues on LSM14A, promoting its accumulation in PBs and supporting their assembly (Fig. 3E) [190].

### Non-classical functions of PRMTs: beyond methyltransferase activity

Growing evidence indicates that PRMTs can regulate cellular processes independently of their canonical arginine methyltransferase activity. For example, PRMT1 forms a transcriptional repressive complex with the unliganded progesterone receptor and its co-regulators (HP1γ, LSD1, and H3K9me3), targeting the promoters of progesterone-responsive genes to suppress their transcription [191]. Notably, pharmacological inhibition of PRMT1’s enzymatic activity does not affect the assembly or function of this complex, highlighting a methylation-independent regulatory role [191]. PRMT1 also interacts with the nuclear receptor testicular receptor 3 (TR3), preventing its association with E3 ubiquitin ligases and thereby inhibiting TR3 ubiquitination and degradation, independent of its catalytic activity [192]. Similarly, PRMT3 represses retinoic acid signaling via a non-enzymatic mechanism by directly inhibiting the catalytic activity of ALDH1A1 [193]. A particularly striking case is PRMT8, which harbors a histidine–lysine–aspartate (HKD) motif in addition to its methyltransferase domain. This motif endows PRMT8 with intrinsic phospholipase activity, enabling it to function both as a methyltransferase and a lipid-modifying enzyme [194].

## Upstream regulatory networks of PRMTs

Although PRMTs are key regulators of gene expression, their own expression and enzymatic activity are themselves subject to multilayered regulation by diverse upstream mechanisms—including transcriptional control, alternative splicing, mRNA stability and translation, PTMs, and metabolite sensing—ensuring precise and context-dependent modulation. PRMTs share some common regulatory features, yet individual members are uniquely controlled. Dysregulation of these pathways is implicated in cancer, neurological disorders, and immune diseases, underscoring the therapeutic potential of targeting both PRMTs and their upstream regulators.

### Transcriptional control of PRMT expression

The transcriptional of PRMTs involves coordinated control by specific transcription factors, chromatin remodeling complexes, and histone modifications (Fig. 4A). Transcription factors such as STAT1, Myc, P53, HNF4A, and NF-κB directly bind to the promoters of different PRMT members to regulate their expression [136, 195–200]. In addition, histamine represses PRMT1 transcription initiation through SWI/SNF-mediated chromatin remodeling [201], while NAA40-dependent N-terminal acetylation of histone H4 may modulate PRMT5 expression by influencing transcription factor binding [202].

### Regulation of PRMTs via Pre-mRNA splicing

Alternative splicing of PRMTs shapes tissue-specific expression and functional diversity, with aberrant events often linked to tumorigenesis (Fig. 4B). PRMT1, the most extensively studied, generates multiple isoforms: v1-v3 are broadly expressed; v4 is heart-specific; v5 is enriched in the pancreas; and v7 is abundant in the heart and skeletal muscle [20, 203]. In breast cancer, PRMT1-v2 is often overexpressed, promoting cellular invasiveness [204]. RNAi screening identified SNW1 and RALY as splicing regulators, whose knockdown alters PRMT1-v2 at both mRNA and protein levels [204]. PRMT4 is regulated by RBM5, which induces exon 9 skipping and nonsense-mediated decay [205], while ESRP1 modulates splicing to generate PRMT4-FL and PRMT4-ΔE15 isoforms that suppress the TGF-β/Smad pathway and reverse chemotherapy resistance in small cell lung cancer [206]. PRMT5 splicing is competitively regulated by SRSF3 and hnRNPH1 at distinct sites in intron 2 and exon 4 [207]. Radiotherapy suppresses SRSF3 expression, increasing the PRMT5-ISO5 isoform and enhancing radiosensitivity [207]. For PRMT7, the splicing quantitative trait locus rs61746794 promotes exon 16 inclusion, increasing the canonical PRMT7-V2 isoform, which more potently drives CRC cell growth and xenograft formation than PRMT7-V1 [208].

### Regulation of PRMT mRNA stability and translation

#### m^6^A RNA methylation

m^6^A methylation plays a pivotal role in modulating PRMT mRNA stability and expression. METTL3, in concert with the m^6^A reader IGF2BP1, enhances m^6^A modification of PRMT5 and PRMT7 transcripts, stabilizing their mRNAs and upregulating protein levels (Fig. 4C). This contributes to activation of the Wnt/β-catenin pathway and progression of triple-negative breast cancer (TNBC) [209, 210]. Similarly, IGF2BP2 stabilizes PRMT6 mRNA in an m⁶A-dependent manner, thereby enhancing PRMT6 expression and increasing H3R2me2a levels [211]. In contrast, the m^6^A demethylase ALKBH5 removes methyl groups from PRMT6 mRNA, promoting its degradation and serving as a negative regulator (Fig. 4C) [212].

#### Non-coding RNAs (ncRNAs)

A network of ncRNAs—including miRNAs, lncRNAs, and circRNAs—orchestrates PRMT expression by regulating transcription, mRNA stability, and translation (Fig. 4C) [213, 214]. Several miRNAs target the 3’ UTRs of PRMT transcripts to suppress translation or promote degradation. For instance, miR-503, miR-574-3p, miR-455-5p, and miR-494-3p target PRMT1 [215–218]; miR-15a, miR-195, miR-16-5p, miR-223, and miR-181c target PRMT4 [219–223]; and miR-103a-3p, miR-92b and miR-96 bind to PRMT5 [47, 224]; and miR-24-2 targets PRMT7 [225]. LncRNAs and circRNAs can indirectly modulate PRMT expression by sponging miRNAs (Fig. 4C). For instance, lncRNA LINC00515 sequesters miR-16, releasing its inhibition on PRMT5 and promoting glioma progression [226]. Similarly, circHMGB2 sponges miR-181a-5p, preventing repression of PRMT4, which promotes immune exhaustion and modulates the tumor microenvironment [227]. Other circRNAs—circLRP6, circZNF532, circRHOT1, circ_0039960, and circ_001726—upregulate PRMT1, PRMT4, PRMT5, PRMT7, and PRMT9, respectively, through competitive binding to their targeting miRNAs [228–232].

#### Autophagy

Autophagy selectively degrades mRNAs by facilitating the interaction of LC3B with target transcripts. Before autophagosome formation, LC3B recruits the CCR4–NOT complex to these mRNAs, triggering rapid deadenylation and degradation [233, 234]. Notably, PRMT1 itself is targeted in this manner: LC3B binds to an AAUAAA motif in PRMT1’s 3’ UTR, promoting transcript degradation and thereby facilitating autophagy (Fig. 4C) [233].

#### Other mechanisms

Additional regulatory mechanisms also influence PRMT mRNA stability and translation. The SMN protein, best known for its role in snRNP assembly and RNA granule dynamics in motor neuron axons, has also been implicated in translational regulation. Evidence suggests that SMN may suppress PRMT4 translation by binding to its coding sequence, possibly in coordination with additional regulatory cofactors (Fig. 4C) [235]. Additionally, hnRNPF, regulated by a super-enhancer, binds to the 3’ UTR of PRMT1 mRNA to stabilize the transcript and prevent degradation [236].

### Post-translational modifications of PRMTs

#### Phosphorylation

Phosphorylation, a reversible modification primarily occurring on serine, threonine, and tyrosine residues, is a key regulator of protein function [237]. Emerging evidence indicates that phosphorylation of PRMTs significantly influences their enzymatic activity, substrate specificity, and subcellular localization.

For PRMT1, phosphorylation at serine/threonine residues modulates its substrate preference, transcriptional regulatory activity, and nuclear-cytoplasmic distribution [238–241]. For example, Tyr291 phosphorylation shifts PRMT1 substrate selectivity toward non-histone peptides over histone H4 [238]. In keratinocytes, CSNK1A1-mediated phosphorylation of PRMT1 promotes proliferation-related gene expression while suppressing differentiation markers (Fig. 4D) [239]. DNA-dependent protein kinase (DNA-PK) also mediates the phosphorylation of PRMT1, facilitating its recruitment to chromatin and reprogramming its enzymatic specificity toward H4R3 [240]. Moreover, CDK5-mediated phosphorylation at Ser307 promotes PRMT1 translocation to the cytoplasm and lysosomes, enabling methylation of WDR24 and subsequent activation of the mTORC1 pathway [241].

PRMT4 is likewise regulated by phosphorylation at multiple sites, with distinct modifications exerting differential effects. Phosphorylation at Ser228 and Ser448 inhibits its methyltransferase activity [242–244], whereas phosphorylation at Tyr149 and Tyr334 enhances activity and alters substrate specificity [245]. In addition, phosphorylation at Ser217, Ser572, and Ser595 promotes cytoplasmic translocation [246–248].

The activity and localization of PRMT5 are modulated by several kinases. RhoA-activated kinase, myosin phosphatase, and PLK4 influence its enzymatic activity through phosphorylation. Conversely, JAK2 V617F-induced phosphorylation at Tyr297, Tyr304, and Tyr307 disrupts the PRMT5–MEP50 complex, inhibiting function [249]. C-terminal threonine phosphorylation by AKT and SGK kinases shifts PRMT5 interactions toward 14-3-3 proteins over PDZ domains, altering both localization and activity [250]. In response to IL-1β, PRMT5 phosphorylation at Ser15 by PKCι is crucial for NF-κB activation [251].

#### Ubiquitination

Ubiquitination regulates protein degradation, localization, and activity by conjugating ubiquitin to lysine residues [252, 253]. E3 ligases such as CHIP, TRIM48, and FBXL17 target PRMT1 for proteasomal degradation (Fig. 4D) [254–257]. Conversely, mechanisms such as TK1/KTN1 interference with TRIM48, lysine acetylation in the IKxxxIK motif, and the activity of LINC01431 or circTBC1D14 stabilize PRMT1 by preventing ubiquitination [258, 259]. Furthermore, USP11 activates PRMT1 by removing its K63-linked ubiquitin chains, demonstrating a role for ubiquitination in directly regulating methyltransferase activity beyond protein stability [260].

In addition to PRMT1, other PRMTs are also subject to ubiquitin-mediated regulation, influencing their stability and function. PRMT4 degradation is mediated by the SCF^FBXO9^ complex and Skp2 [261–263], while TRIM28 and the lncRNA ST7-AS1 block E3 ligase interactions to stabilize PRMT4 [264, 265]. Phosphorylation by GSK-3β at Thr132 also protects PRMT4 from ubiquitin-mediated degradation [266]. PRMT5 stability is maintained via its interaction with LINC01138, which suppresses its ubiquitination [267]. PRMT6 is targeted for degradation by FBXW17 and FBXO24, but phosphorylation at Ser11 and Thr21 by CK2 promotes its stabilization [268].

#### Arginine methylation

PRMTs undergo auto-methylation at specific arginine residues, influencing their activity and stability (Fig. 4D). For instance, PRMT4 is capable of self-methylation at the R551 site in its C-terminal region, independent of other methyltransferases [269]. PRMT6 predominantly auto-methylates at R35, and mutations at this site impair both its methylation capacity and stability [43]. PRMT7’s tandem methyltransferase modules enable intermolecular auto-methylation, critical for its activity and promotion of epithelial–mesenchymal transition (EMT), migration, and invasion [270]. PRMT8 also contains two auto-methylation sites and an N-terminal myristoylation signal. While myristoylation facilitates membrane association, auto-methylation may suppress its enzymatic activity by altering the conformation of the AdoMet-binding pocket or reducing its binding affinity for the methyl donor [41, 271]. In addition to self-methylation, PRMTs can be methylated by other family members (Fig. 4D). PRMT4 methylates PRMT5 at R505, promoting oligomerization while suppressing its methyltransferase activity—essential for PRMT5 homodimer formation and catalytic regulation [272].

#### Other PTMs

Additional PTMs—including acetylation, O-GlcNAcylation, succinylation, S-nitrosylation, and glutathionylation—further fine-tune PRMT localization, stability, substrate specificity, and enzymatic activity (Fig. 4D) [273–277]. In CRC, LPCAT2-mediated acetylation of PRMT1 at K145 inhibits its nuclear translocation [273]. O-GlcNAcylation of PRMT4 alters its substrate preference without affecting its overall stability [274]. SIRT7-mediated desuccinylation of PRMT5 enhances octamer assembly and catalytic activity, while preventing STUB1-mediated degradation [275]. S-nitrosylation of PRMT1 at C119 under oxidative stress impairs its activity [276]. Similarly, glutathionylation of PRMT5 at Cys42 disrupts MEP50 binding and reduces enzymatic function, which can be reversed by Grx1 [277, 278].

### Interplay between metabolism and PRMT activity

The enzymatic activity of PRMTs is governed not only by their expression but also by metabolic cues within the microenvironment, particularly the intracellular AdoMet/AdoHcy ratio and methylthioadenosine (MTA) levels (Fig. 4E). AdoMet functions as the principal methyl donor for arginine methylation, whereas AdoHcy, its byproduct, acts as a competitive inhibitor. A reduced AdoMet/AdoHcy ratio—commonly observed under nutrient deprivation—can markedly impair PRMT catalytic activity [14]. AdoMet availability is sustained through the methionine cycle, in which methylthioadenosine phosphorylase (MTAP) degrades MTA, a metabolite that otherwise accumulates and suppresses PRMT activity. Thus, MTAP plays a critical role in maintaining methylation capacity by limiting MTA-mediated inhibition of PRMTs.

## PRMTs and the hallmarks of cancer

**Fig. 5:**
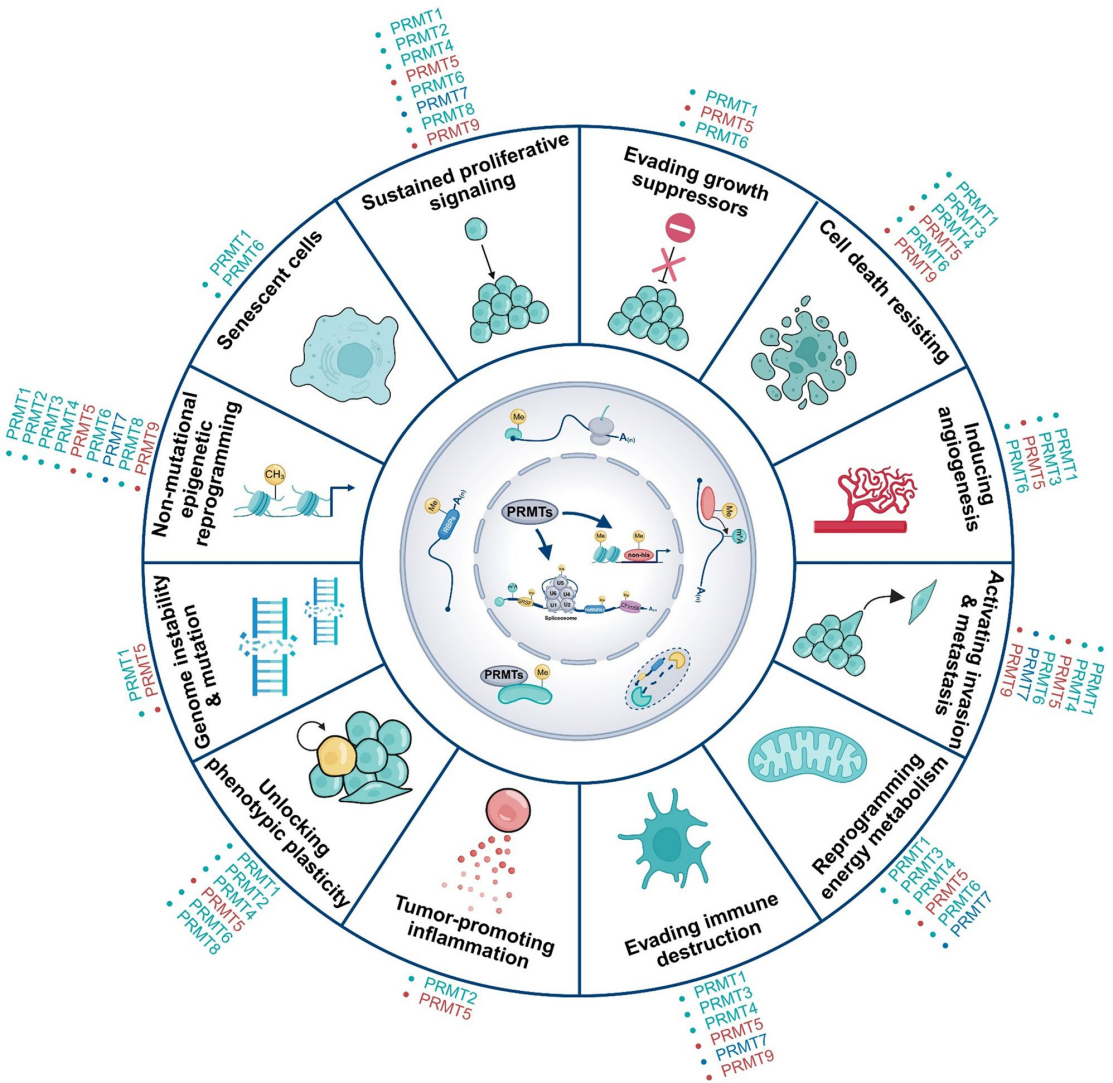
Regulatory Roles of PRMTs in the Hallmarks of Cancer. PRMTs contribute to the acquisition of cancer hallmarks by orchestrating transcriptional and post-transcriptional regulation, as well as modulating key signaling pathways. These hallmarks include: sustained proliferative signaling, evasion of growth suppressors, resistance to cell death, induction of angiogenesis, activation of invasion and metastasis, reprogramming of energy metabolism, immune evasion, tumor-promoting inflammation, acquisition of phenotypic plasticity, genome instability and mutation, non-mutational epigenetic reprogramming, and regulation of senescent cells

The concept of cancer hallmarks, articulated by Hanahan and Weinberg, provides a unifying framework to describe the biological capabilities acquired during tumor progression [279–281]. In this section, we summarize recent insights into the roles of PRMTs in cancer biology and how they contribute to the acquisition and maintenance of hallmark traits and enabling characteristics (Fig. 5; Table 1). Notably, these functions operate not in isolation but as part of a dynamic and interconnected network that collectively drives tumor progression.

**Table 1:** Regulatory roles of PRMTs in the hallmarks of cancer

Cancer hallmarks	PRMTs	Target (Mechanism)	Cancer	Ref
Sustained proliferative signaling				
	PRMT1	Methylation (EGFR)	CRC	[283]
		Methylation (C/EBPα, EZH2)	Breast cancer	[115] [284]
		Altered expression (β-catenin)	PDAC	[618]
		Formation of the translation initiation complex	Osteosarcoma	[180]
		Methylation (WDR24, PHGDH)	Liver cancer	[241, 286]
	PRMT2	Methylation (H3R8)	Glioblastoma	[39]
		Methylation (H3R8)	RCC	[289]
	PRMT4	ERα-mediated transcriptional activation coordination	Breast cancer	[291]
		Methylation (H3R17)	Breast cancer	[292]
		Transcriptional coactivator (ACTR)	Breast cancer	[293]
	PRMT5	Methylation (H4R3)	Gastric cancer	[121]
		Methylation (KLF4)	Breast cancer	[296]
		Methylation (PDCD4))	Breast cancer	[297]
		Methylation (H4R3, H3R8)	CRC	[61]
		Altered expression (PTEN)	Glioblastoma	[298]
		Methylation (H4R3)	Lung cancer	[300]
		Altered phosphorylation (ERK)	Liver cancer	[301]
		Methylation (TRIM21)	Multiple myeloma	[302]
	PRMT6	Formation of the transcription-repressive complex (PARP1, CRL4B)	Breast cancer	[303]
		Methylation (RCC1)	Glioblastoma	[268]
		Activation (ERK, mTOR)	EC	[304]
	PRMT7	Methylation (PTEN)	Gastric cancer	[305]
		Methylation (β-catenin)	RCC	[306]
	PRMT9	Activation (Notch)	Liver cancer	[231]
Evading growth suppressors				
	PRMT1	Altered expression (P53)	Retinoblastoma	[314]
	PRMT5	Methylation (P53)	Leukemia/lymphoma	[311]
		Methylation (H3R8, H4R3)	Leukemia/lymphoma	[317]
		Altered expression (eIF4E)	Breast cancer	[312]
	PRMT6	Altered expression (P21)	Breast cancer	[315]
Cell death resisting				
	PRMT1	Methylation (HSP70)	PDAC	[169]
		Methylation (p14ARF)	PDAC	[329]
		Methylation (E2F1)	Osteosarcoma	[122]
		Methylation (RIPK3)	CRC	[336]
		Altered cofactor binding (SGs)	Breast cancer	[259]
		Altered expression (ACSL1)	AML	[347]
	PRMT3	Methylation (METTL14)	EC	[173]
	PRMT4	Altered expression (Nrf2)	NPC	[349]
	PRMT5	Methylation (E2F1)	Osteosarcoma	[332]
		Methylation (ULK1)	Cancers	[341]
		Methylation (KEAP1)	Breast cancer	[350]
		Methylation (GPX4)	Cancers	[351]
		Methylation (H4R3)	ESCC	[352]
	PRMT6	Methylation (BAG5)	Liver cancer	[340]
	PRMT9	Methylation (HSPA8)	Liver cancer	[356]
Inducing angiogenesis				
	PRMT1	Activation (STAT3)	Liver cancer	[365]
	PRMT3	Methylation (HIF-1α)	CRC	[364]
	PRMT5	Methylation (H3K4)	Retinoblastoma	[362]
		Methylation (H3R2)	Glioblastoma	[363]
		Altered expression (HIF-1α)	Lung cancer	[367]
		Methylation (H3R2)	Liver cancer	[370]
	PRMT6	Methylation (H3R2)	Osteosarcoma	[373]
		Altered expression (TSP-1, MMPs)	Prostate cancer	[374]
Activating invasion and metastasis				
	PRMT1	Methylation (EZH2)	Breast cancer	[60]
		Methylation (Twist1)	Lung cancer	[116]
		Methylation (H4R3)	Breast cancer	[390]
		Altered expression (ZEB1)	PDAC	[391]
		Methylation (PGC-1α)	NPC	[404]
		Methylation (HBP1)	Cancers	[406]
	PRMT4	Methylation (LSD1)	Breast cancer	[384]
		Cofactor binding (RPF2)	CRC	[394]
	PRMT5	Formation of the transcription-repressive complex (ZEB2, TWIST1, NuRD)	CRC	[385]
		Methylation (H4R3, H3R2)	Breast cancer	[388]
		Formation of the transcription-repressive complex (Snail, NuRD)	Cervical cancer	[389]
		Methylation (H3R2)	HNSCC	[392]
		Activation (Wnt)	Laryngeal carcinoma	[395]
		Activation (nucleolin)	Prostate cancer	[396]
		Methylation (AKT)	Neuroblastoma	[398]
		Methylation (H4R3)	Lung cancer	[300]
		Methylation (H3R2)	Bladder cancer	[49]
		Altered expression (LKB1)	ESCC	[401]
		Methylation (MTHFD1)	ESCC	[405]
		Cofactor binding (IRX1)	Gastric cancer	[407]
	PRMT6	Methylation (STAT3)	Breast cancer	[408]
	PRMT7	Methylation (H4R3)	Breast cancer	[386]
		Automethylation	Breast cancer	[270]
	PRMT9	Activation (PI3K)	Liver cancer	[397]
Reprogramming energy metabolism				
	PRMT1	Methylation (PFKFB3)	Liver cancer	[255]
		Methylation (PGK1)	CRC	[419]
		Methylation (PHGDH)	Breast cancer; Liver cancer	[443] [286]
	PRMT3	Methylation (GAPDH)	Pancreatic cancer	[416]
		Methylation (LDHA)	Liver cancer	[424]
		Altered expression (HIF1A)	Glioblastoma	[425]
	PRMT4	Methylation (GAPDH)	Liver cancer	[417]
		Methylation (PKM2)	Breast cancer	[422]
		Altered expression (YAP, H3R17me)	Osteosarcoma	[426]
		Methylation (H3R17)	Gastric cancer	[430]
		Methylation (RPIA)	CRC	[431]
		Methylation (MDH1)	PDAC	[439]
	PRMT5	Altered expression (FBW7)	Pancreatic cancer	[428]
		Altered expression (LXRα)	Breast cancer	[427]
		Methylation (SREBP1a)	Cancers	[275, 619]
		Methylation (G3BP2)	HNSCC	[434]
	PRMT6	Methylation (6PGD, ENO1)	Lung cancer	[421]
		Methylation (CRAF)	Liver cancer	[423]
	PRMT7	Methylation (H2AR3)	CML	[445]
Evading immune destruction				
	PRMT1	Methylation (cGAS)	Cancers	[456]
		Methylation (H4R3)	Melanoma	[459]
		Altered expression (PD-L1,PD-L2)	Liver cancer	[465]
		Methylation (PGC-1α)	NPC	[404]
	PRMT3	Methylation (HSP60)	Liver cancer	[453]
	PRMT4	Methylation (BAF155)	Breast cancer	[462]
	PRMT5	Methylation (IFI16); Altered expression (NLRC5)	Melanoma	[457]
		Methylation (PD-L1)	Lung cancer	[467]
		Methylation (H3R2)	Cervical cancer	[466]
		Methylation (H4R3, H3R2)	Prostate cancer	[457]
	PRMT7	Altered expression (DNMT, RIG-I, MDA5)	Melanoma	[460]
	PRMT9	Methylation (PABPC1)	AML	[454]
Tumor-promoting inflammation				
	PRMT2	Inhibition (NF-κB, STAT3)	AML	[484]
	PRMT5	Methylation (NF-κB)	CRC	[124]
		Methylation (YBX1)	CRC	[485]
Unlocking phenotypic plasticity				
	PRMT1	Methylation (SOX2)	Lung cancer	[493]
		Cofactor binding (MLXIP, KTN1)	Gastric cancer	[256]
		Methylation (H4R3)	Liver cancer	[509]
		Methylation (DDX3)	Breast cancer	[510]
	PRMT2	Methylation (H3R8)	RCC	[289]
	PRMT4	Cofactor binding (HIF1A)	Breast cancer	[295]
	PRMT5	Promotion (Stemness)	Breast cancer	[494]
		Methylation (H3R2)	Breast cancer	[495]
		Methylation (KLF5)	Breast cancer	[496]
		Methylation	CML	[502]
		Inhibition (Stemness)	Gastric cancer	[503]
	PRMT6	Altered expression (YTHDF2)	Glioblastoma	[177]
		Methylation (CRAF)	Liver cancer	[506]
	PRMT8	Promotion (Stemness)	CRC	[498]
Genome instability and mutation				
	PRMT1	Methylation (MRE11, 53BP1)	Cancers	[520]
		Methylation (hnRNPUL1)	Cancers	[522]
		Methylation (BRCA1)	Breast cancer	[525]
		Methylation (APE1)	Cervical cancer	[534]
		Altered expression (FEN1)	Lung cancer	[537]
	PRMT5	Methylation (ALKBH7)	Breast cancer	[527]
		Methylation (53BP1)	Cancers	[528]
		Methylation (RUVBL1)	Cancers	[106]
		Methylation (FEN1)	Cervical cancer	[538]
Non-mutational epigenetic reprogramming				
	PRMT1	Methylation (H4R3)	CML	[35]
		Methylation (C/EBPα )	Breast cancer	[115]
		Methylation (c-Fos )	Gastric cancer	[117]
		Methylation (Gli1)	PDAC	[119]
		Methylation (SRSF1 )	ALL	[151]
		Methylation (METTL14)	Cancers	[172]
		Methylation (WTAP)	Multiple myeloma	[176]
		Methylation (UBAP2L)	Cancers	[188, 236]
		Methylation (RAP55A)	Cervical cancer	[190]
		Formation of the transcription-repressive complex (uPR)	Breast cancer	[191]
		Altered expression (TR3)	Liver cancer	[192]
	PRMT3	Methylation (hnRNPA1)	Pancreatic cancer	[154]
	PRMT4	Methylation (BAF155)	Breast cancer	[92]
		Methylation (BAF155)	Ovarian cancer	[93]
		Methylation (GATAD2A/2B)	Breast cancer	[98]
		Methylation (NONO)	Cancers	[168]
	PRMT5	Methylation (H4R3)	Cancers	[44, 46]
		Methylation (H4R3, H3R8); Cofactor binding (HIF1A)	Lymphoid cancer	[47]
		Methylation (H4R3)	Cervical cancer	[77]
		Cofactor binding (UHRF1)	EC	[81]
		Methylation (MBD)	Cancers	[97]
		Cofactor binding (FUS)	Breast cancer	[113]
		Methylation (Gli1)	Cancers	[120]
		Cofactor binding (SMN complex)	Cervical cancer	[132]
		Methylation (Sm)	Cancers	[135, 137, 147]
		Methylation (hnRNPA1)	Cancers	[145]
		Methylation (METTL14)	AML	[174]
	PRMT6	Methylation (H3R2)	Cancers	[83]
		Methylation (ALKBH5)	Breast cancer	[175]
	PRMT7	Methylation (hnRNPA1)	Cancers	[153]
		Methylation (NUDT21)	Prostate cancer	[158]
	PRMT8	Hydrolysis (Phosphatidylcholine)	Pheochromocytoma	[194]
	PRMT9	Methylation (SAP145)	Cervical cancer	[134]
Senescent cells				
	PRMT1	Methylation (H4R3)	Breast cancer Ovarian cancer	[390] [240]
	PRMT6	Methylation (H3R2)	Breast cancer	[315]

ALL, acute lymphoblastic leukemia; AML, acute myeloid leukemia; CML, chronic myeloid leukemia; CRC, colorectal cancer; EC, endometrial cancer; ESCC, esophageal squamous cell carcinoma; HNSCC, head and neck squamous cell carcinoma; NPC, nasopharyngeal carcinoma; PDAC, pancreatic ductal adenocarcinoma; RCC, renal cell carcinoma

### Sustained proliferative signaling

One of the defining hallmarks of cancer is the ability of tumor cells to sustain proliferative signaling by hijacking key growth regulatory pathways [282]. PRMTs emerge as core regulators of this process in diverse malignancies, where they modulate a multi-layered regulatory cascade—encompassing transcription, post-transcriptional modification, and signal transduction—to reinforce oncogenic proliferation (Fig. 5).

PRMT1 promotes proliferation by targeting multiple signaling nodes. In CRC, it methylates EGFR at R198/R200 in the extracellular domain, enhancing ligand binding and receptor activation to drive tumor cell proliferation [283]. In breast cancer, PRMT1 modifies C/EBPα and EZH2 to activate Cyclin D1 while repressing p16 and p21, accelerating G1/S progression [115, 284]. In pancreatic ductal adenocarcinoma (PDAC), PRMT1 supports tumor growth by regulating RNA metabolism, cell cycle genes, and β-catenin signaling [285]. Additional studies further demonstrate that PRMT1 drives tumor proliferation through distinct mechanisms, including regulation of translation in osteosarcoma, activation of mTORC1 signaling in hepatocellular carcinoma, and transcriptional control of cell-cycle progression in glioma and multiple myeloma [180, 286–288].

PRMT2 acts as an oncogene driver in several cancers by modulating transcription and signaling. In glioblastoma, PRMT2 overexpression predicts poor prognosis, whereas its loss suppresses tumor growth and stemness by downregulating oncogenic and cell cycle genes through H3R8me2a-dependent promoter and enhancer activation [39]. In clear cell renal cell carcinoma, PRMT2 promotes WNT5A expression via H3R8me2a on its promoter, activating non-canonical Wnt signaling and driving proliferation and migration [289, 290].

PRMT4 is a key regulator of hormone- and stress-responsive transcriptional programs. In ER-positive breast cancer, it is recruited by ERα to the promoters and enhancers of cell cycle genes, catalyzing H3R17me2a at the E2F1 promoter to drive expression of E2F1 and downstream cyclins such as CCNE1/2 and CCNA1 [291, 292]. It also cooperates with ACTR, a p160 coactivator, to potentiate estrogen-induced transcription [293]. In prostate cancer, PRMT4 enhances androgen receptor (AR) signaling via histone methylation at AR target genes, contributing to tumor progression [294]. Under hypoxia, PRMT4 is induced by HIF-1α and occupies promoters of CDK4, Cyclin D1, β-Catenin, MALAT1, and SIX1, thereby activating HIF-1, Wnt, and VEGF pathways to promote TNBC proliferation and invasion [295].

PRMT5 facilitates cell proliferation by repressing tumor suppressors and activating oncogenic networks. In gastric cancer, PRMT5-mediated H4R3me2s overlaps with c-Myc binding regions to silence genes such as PTEN, CDKN2C, and CDKN1A [121]. In breast cancer, PRMT5 promotes tumor growth by methylating key substrates: it stabilizes KLF4 by inhibiting its ubiquitylation, leading to Bax downregulation and oncogene upregulation, and it methylates PDCD4, abolishing its tumor-suppressive function [296, 297]. In CRC, PRMT5 cooperates with EZH2 to silence CDKN2B, accelerating cell-cycle progression [61]. In glioblastoma, it represses ST7 and other tumor suppressors to maintain proliferation and stem-like features [298, 299]. In lung cancer, PRMT5 downregulates miR-99 family, elevating FGFR3 and activating ERK/AKT signaling to promote tumor growth [300]. In HCC, PRMT5 suppresses BTG2 via ERK to promote G1/S transition [301]. In multiple myeloma, PRMT5 inhibition destabilizes IKKβ via TRIM21, blocking NF-κB signaling and reducing proliferation [302].

PRMT6 contributes to tumor proliferation by regulating transcription and mitotic signaling. In breast cancer, it forms a repressive complex with PARP1 and CRL4B to silence PER3, disrupting circadian regulation and promoting growth and metastasis [303]. In glioblastoma, PRMT6 methylates RCC1 at R214, enhancing its chromatin binding, promoting mitosis, stem cell expansion, and drug resistance [268]. In endometrial cancer, PRMT6 is upregulated and drives oncogenic progression by activating the AKT/mTOR pathway [304].

Other PRMTs also contribute to tumor growth through diverse mechanisms. In gastric cancer, PRMT7 interacts with PTEN, promoting its arginine methylation and expression, thereby suppressing downstream PI3K/AKT signaling and inhibiting cell proliferation and migration [305]. In clear cell renal cell carcinoma, PRMT7 methylates β-catenin, preventing its ubiquitin-mediated degradation, which leads to c-Myc upregulation and accelerated tumor growth [306]. PRMT7 also regulates the growth of breast cancer and liver cancer stem cells [209, 225, 230]. PRMT8 is essential for the proliferation of high-grade glioma cells, though its mechanism of action has yet to be elucidated [307]. In hepatocellular carcinoma, PRMT9 expression activating the Notch pathway and enhancing malignant phenotypes, including increased proliferation [231].

### Evading growth suppressors

To sustain uncontrolled proliferation, cancer cells frequently bypass growth-inhibitory mechanisms governed by core tumor suppressors such as p53, p21/CDKN1A, and RB [308], with PRMTs facilitating this evasion by modulating these pathways and compromising intrinsic growth checkpoints (Fig. 5). p53 functions as a central tumor suppressor and transcription factor that regulates genes involved in cell cycle arrest, apoptosis, senescence, and DNA repair [309–311]. In lymphoma, PRMT5 directly methylates p53, selectively repressing its transcriptional activation of pro-apoptotic and anti-proliferative genes—thus promoting tumor cell self-renewal even in the absence of p53 mutations [311]. In breast cancer, PRMT5 has been shown to modulate p53 translation through regulation of eIF4E, influencing p53’s tumor-suppressive activity and highlighting context-dependent effects [312]. The canonical p53 downstream effector p21 acts as a cyclin-dependent kinase inhibitor to enforce G1 arrest and induce senescence [313]. PRMT1 overexpression in retinoblastoma promotes proliferation, likely through the p53/p21/CDK1/Cyclin B axis [314]. In contrast, PRMT6 represses p21 expression through direct methylation of its promoter independently of p53. Silencing PRMT6 in breast cancer cells derepresses p21, inducing cell cycle arrest, senescence, and reduced tumor growth [315]. RB, the first tumor suppressor gene identified in humans, governs cell cycle progression by modulating E2F transcription factors [316]. In leukemia and lymphoma, PRMT5 silences RB family members (RB1, RBL1, and RBL2) via hypermethylation of H3R8 and H4R3, thereby disabling RB-mediated cell cycle control and promoting proliferation [317].

### Cell death resisting

Programmed cell death (PCD), governed by distinct molecular mechanisms, is a critical barrier to tumorigenesis and a key determinant of therapeutic efficacy [318–320]. Among the major PCD subtypes—apoptosis [321, 322], necroptosis [323], autophagic cell death [324], and ferroptosis [325]—PRMTs have emerged as key modulators. By regulating these pathways, PRMTs enable cancer cells to evade cell death and sustain survival under various stress conditions (Fig. 5).

**Fig. 6:**
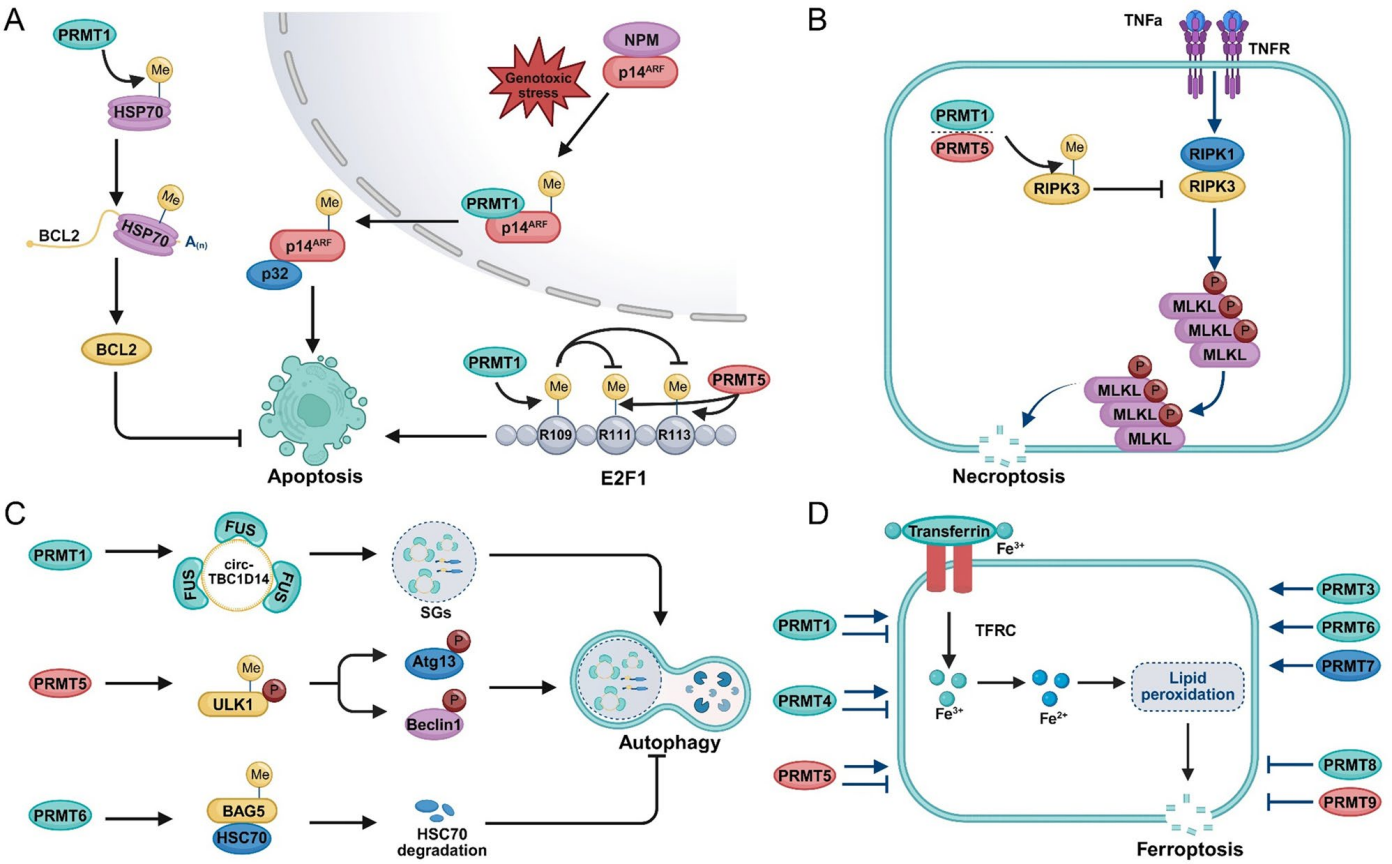
Roles of PRMTs in Resisting Cell Death. (**A**) PRMTs regulate apoptosis through multiple mechanisms. PRMT1 methylates HSP70 to stabilize BCL2 mRNA and modifies p14^ARF^ to control its subcellular localization. Both PRMT1 and PRMT5 also methylate distinct arginine residues on E2F1, modulating its transcriptional activity. (**B**) PRMT1 and PRMT5 influence necroptosis by regulating RIPK3 methylation. (**C**) PRMTs modulate autophagy by promoting FUS–circTBC1D14 complex formation and methylating autophagy-related proteins such as ULK1 and BAG5. (**D**) Different PRMTs exhibit either pro- or anti-ferroptotic effects, depending on the cellular context

Apoptosis is the most classical form of non-inflammatory PCD, characterized by a caspase-dependent self-clearance mechanism [326, 327]. In response to physiological or pathological stimuli such as DNA damage or oxidative stress, cells initiate a cascade of molecular events through either the intrinsic pathway—marked by changes in mitochondrial membrane permeability—or the extrinsic pathway, involving activation of death receptors. These processes ultimately lead to cell shrinkage, chromatin condensation, and apoptotic body formation, which are phagocytosed without triggering an inflammatory response [328]. The BCL-2 family serves as a central regulator of the intrinsic apoptotic pathway, encompassing both pro- and anti-apoptotic members [322]. In PDAC, PRMT1 promotes HSP70 methylation, which stabilizes BCL2 mRNA and elevates BCL-2 protein levels, thereby suppressing apoptosis and conferring chemoresistance (Fig. 6A) [169]. The CDKN2A gene encodes two critical tumor suppressors: p14^ARF^ and p16^INK4a^ [329]. Among them, p14^ARF^ can induce cell cycle arrest and apoptosis through both p53-dependent and independent pathways [330]. Under cellular stress, PRMT1 methylates the C-terminal nuclear localization sequence of p14^ARF^, promoting its redistribution between the nucleus and cytoplasm, thereby facilitating p53-independent apoptosis (Fig. 6A) [329]. E2F1 is a pivotal transcription factor that governs both cell proliferation and apoptosis. Its cellular concentration dictates cell fate: low levels promote proliferation, moderate levels induce cell cycle arrest, and high levels trigger apoptosis [331]. PRMT5 reduces E2F1 stability via arginine methylation at residues R111 and R113 [332]. Conversely, PRMT1-mediated methylation of E2F1 at R109 inhibits PRMT5 binding, shifting the balance toward E2F1-dependent apoptosis (Fig. 6A) [122].

Necroptosis is a form of PCD that is initiated by stimuli similar to those triggering apoptosis but is morphologically distinct, resembling necrosis with features such as organelle swelling and plasma membrane rupture [333]. The canonical death receptor–mediated necroptosis pathway involves a signaling cascade composed of RIPK1, RIPK3, and MLKL, which is activated downstream of tumor necrosis factor receptors (e.g., TNFR, Fas) and Toll-like receptors 3/4 (TLR3/4) [334, 335]. PRMT1 suppresses necroptosis by methylating RIPK3 at a conserved arginine residue (R486 in humans, R479 in mice), thereby disrupting its interaction with RIPK1 (Fig. 6B). This inhibition prevents the formation of the RIPK1–RIPK3 complex and subsequent RIPK3 phosphorylation, ultimately blocking necroptosis activation [336]. Additionally, PRMT5 has also been shown to methylate RIPK3 (R486 in humans, R415 in mice), contributing to the negative feedback regulation of RIPK3 activity and its interaction with RIPK1 (Fig. 6B) [337].

Tumor cells often reside in microenvironments characterized by hypoxia, nutrient deprivation, and various stressors. Autophagy is crucial for maintaining cellular homeostasis by removing damaged organelles and misfolded proteins while supporting biosynthesis under metabolic stress, enabling cancer cells to survive hostile conditions [338–340]. However, under certain circumstances, excessive or dysregulated autophagy can lead to a distinct form of cell death known as autophagic cell death [324]. Under hypoxic conditions, PRMT1 modifies FUS-circTBC1D14, promoting the formation of SGs in TNBC. These SGs recruit LAMP1 to enhance lysosome-associated autophagic flux, triggering a cascade of stress-related protein responses. This facilitates granule clearance and the maintenance of homeostasis, ultimately promoting tumor progression (Fig. 6C) [259]. Concurrently, PRMT5-mediated methylation of ULK1 at R170 promotes T180 phosphorylation, activating autophagy via ATG13 and Beclin1 phosphorylation, which enhances mitochondrial clearance, reduces oxygen consumption, and supports tumor survival and proliferation (Fig. 6C) [341]. In HCC, PRMT6 deletion increases autophagy under hypoxia, nutrient deprivation, or sorafenib-induced stress. Mechanistically, PRMT6 represses autophagy by methylating the co-chaperone BAG5, enhancing its interaction with HSC70, a key autophagy regulator, thereby promoting its degradation and suppressing autophagic activity (Fig. 6C) [340].

Ferroptosis is a distinct form of PCD driven by the iron-dependent accumulation of lipid peroxides, particularly in phospholipids enriched with polyunsaturated fatty acids. It is characterized by dysregulated iron metabolism, excessive lipid peroxidation, and DNA damage [342]. Morphologically, ferroptotic cells exhibit mitochondrial shrinkage, increased membrane density, and reduced or absent cristae—features that distinguish it from other types of PCD [343]. A recent review has systematically summarized the regulatory roles of PRMTs in ferroptosis, and this topic will not be further discussed in the present article [344]. Briefly, the nine known PRMTs can be grouped into three functional categories based on their roles in ferroptosis: dual-function regulators, ferroptosis promoters, and ferroptosis inhibitors. Among them, PRMT1, PRMT4, and PRMT5 exhibit context-dependent, bidirectional regulatory effects—either promoting or inhibiting ferroptosis depending on the cellular environment and stress signals (Fig. 6D) [345–352]. In contrast, PRMT3, PRMT6, and PRMT7 predominantly function as positive regulators of ferroptosis, while PRMT8 and PRMT9 mainly serve inhibitory roles (Fig. 6D) [173, 353–356].

### Inducing angiogenesis

Angiogenesis is a critical process in tumor initiation and progression. By fostering new blood vessel formation, it ensures the supply of oxygen and nutrients required for sustained tumor growth and facilitates metastatic dissemination. Aberrant tumor angiogenesis not only enhances invasiveness but also compromises therapeutic efficacy and promotes treatment resistance [357, 358].

VEGF (VEGFA) is a central driver of angiogenesis, critical for hemangioblast formation during embryogenesis and for generating hematopoietic and endothelial lineages [359]. In tumors, VEGF drives the development of structurally abnormal and functionally immature vasculature [360, 361]. PRMTs regulate VEGF expression either directly or indirectly via upstream transcription factors such as HIF-1α and STAT3 [362–365]. For example, in retinoblastoma, PRMT5 promotes VEGF transcription by enhancing H3K4me3 at its promoter, and in gliomas, it cooperates with HOXC10 and WDR5 to upregulate VEGF expression [362, 363]. Hypoxia-driven neovascularization, largely mediated by HIF-1, is critical for tumor progression through activation of pro-angiogenic genes, including VEGF [366]. In CRC, PRMT3 methylates HIF-1α at R282, stabilizing the protein and enhancing VEGF transcription [364]. Similarly, in lung cancer, PRMT5 inhibition disrupts the HIF-1α/VEGFR/AKT/eNOS axis and downstream nitric oxide production, thereby impairing angiogenesis and EMT [367]. In HCC, PRMT1 activates the STAT3 pathway, upregulating VEGFA, c-Myc, and IL-6 to promote angiogenesis and intrahepatic metastasis [365].

Beyond VEGF/VEGFR signaling, the ANGPT-TIE pathway is another critical regulator of tumor angiogenesis [368, 369]. ANGPTs bind to the endothelial-specific TIE2 receptor and play essential roles in vascular maturation, remodeling, and the angiogenic switch in tumors. In HCC, MYBL1 forms a complex with PRMT5, MEP50, and WDR5 to activate ANGPT2 transcription, thereby promoting angiogenesis and conferring sorafenib resistance [370]. Thrombospondin 1 (TSP-1), a matricellular glycoprotein, functions as an endogenous inhibitor of angiogenesis by engaging multiple receptors and ligands [371, 372]. PRMT6 suppresses TSP-1 transcription by altering H3R2 and H3K4 methylation at its promoter in breast, prostate, and osteosarcoma cells, thereby facilitating tumor angiogenesis [373, 374].

### Activating invasion and metastasis

Metastasis is a multistep process whereby malignant cells disseminate from the primary tumor, enter the circulation, and colonize distant organs [375]. Oncogenes and tumor suppressor genes act in concert to enhance invasiveness, increase vascular permeability, facilitate dissemination, enable adaptation to extracellular matrix and metabolic stress, remodel the metastatic niche, and adapt to heterogeneous microenvironments [376–378]. PRMTs promote metastasis by regulating the transcriptional activity and PTMs of key metastasis-related genes, thereby enhancing the metastatic capacity of cancer cells (Fig. 5; Table 1).

#### EMT

EMT is a biological process by which epithelial cells acquire mesenchymal-like characteristics, playing critical roles in embryonic development, tissue differentiation, wound healing, and tumor metastasis [379, 380]. In cancer, EMT-like cells are enriched at invasive fronts, where they drive migration, invasion, and metastatic dissemination [376, 381]. EMT is regulated at multiple levels, including transcriptional regulation, epigenetic modification, mRNA splicing, translation, and PTMs [382].

A hallmark of EMT is the downregulation of the epithelial cell–cell adhesion molecule E-cadherin (encoded by CDH1), alongside upregulation of mesenchymal markers such as vimentin [383]. PRMTs regulate EMT by modulating these markers via epigenetic mechanisms. For example, PRMT1 and PRMT4 methylate EZH2 (R342) and LSD1/KDM1A (R838), respectively, enhancing their repression of CDH1, thereby suppressing E-cadherin, upregulating vimentin, and promoting EMT and metastasis [60, 384]. PRMT5 promotes CRC metastasis by repressing E-cadherin through interactions with ZEB2/TWIST1/NuRD complexes [385]. In addition, the YY1/PRMT7/HDAC3 complex represses E-cadherin transcription by increasing H4R3me2s levels and reducing H3K4me3, H3Ac, and H4Ac levels [386]. PRMT7 automethylation at R531 further stabilizes this complex [270].

Core EMT transcription factors such as SNAI1/2, TWIST1/2, and ZEB1/2 orchestrate EMT by repressing epithelial markers (e.g., CDH1) and activating mesenchymal genes (e.g., VIM, FN1, CDH2, and FSP1) [387]. In lung cancer, PRMT1 methylates TWIST1 at R34, regulating genes involved in cytoskeletal remodeling and cell motility to promote metastasis [116]. PRMT5 forms a complex with SLUG and LSD1, inducing H4R3me2s and removing H3K4me2 to repress E-cadherin, while activating vimentin transcription via H3R2me2s and H3K9me2 demethylation [388]. In cervical cancer, PRMT5 associates with Snail and the NuRD complex to mediate histone dimethylation and deacetylation, thereby repressing E-cadherin expression and driving EMT [389]. Beyond modulating EMT transcription factor activity, PRMTs also regulate their expression. In breast cancer, PRMT1 promotes ZEB1 transcription via H4R3me2a, facilitating cell migration and invasion [390, 391]. In head and neck squamous cell carcinoma (HNSCC), PRMT5-mediated H3R2me2s facilitates the recruitment of the MLL/SET1/WDR5 complex to the TWIST1 promoter, increasing H3K4me3 and TWIST1 transcription, thereby promoting EMT and lymph node metastasis [392].

EMT is also orchestrated by signaling pathways such as Wnt, PI3K/AKT, and Notch, along with various stimuli including growth factors, cytokines, hypoxia, and extracellular matrix (ECM) remodeling [387, 393, 394]. In laryngeal cancer, PRMT5 activates the WNT4/β-catenin axis to upregulate EMT markers and MMPs, promoting tumor growth and lymph node metastasis [395]. In CRC, prostate cancer, and HCC, PRMT4, PRMT5, and PRMT9 drive EMT via PI3K/AKT signaling [394, 396–398]. Additionally, in lung cancer, PRMT5 suppresses miR-99 through H4R3me2s, upregulating FGFR3 and activating ERK1/2 and AKT pathways to promote tumor progression [300].

#### MMPs

The ECM maintains tissue homeostasis and influences cancer progression through its structural, enzymatic, and biomechanical properties [399]. MMPs degrade ECM components and are critical mediators of invasion and metastasis [400]. In bladder cancer, PRMT5 interacts with HSF1 to induce H3R2me1/2s, recruiting the MLL/WDR5 complex and promoting H3K4me3, thereby upregulating MMP9 and driving lymph node metastasis [49]. In esophageal squamous cell carcinoma, PRMT5 enhances MMP2 and MMP9 expression via the LKB1/AMPK/mTOR axis, promoting tumor growth and metastatic dissemination [401].

#### Anoikis resistance

Anoikis, a form of apoptosis triggered by loss of ECM attachment, is critical for tissue development and homeostasis [402]. Tumor cells that acquire anoikis resistance survive under anchorage-independent conditions, enabling their persistence in circulation and facilitating colonization of distant sites [403]. In nasopharyngeal carcinoma, LMP1 promotes the interaction between PRMT1 and PGC-1α, leading to its methylation, stabilization, and enhanced anoikis resistance, thereby increasing metastatic potential [404]. Additionally, PRMT5 methylates MTHFD1, boosting its metabolic activity and elevating nicotinamide adenine dinucleotide phosphate (NADPH) levels, which contributes to anoikis resistance and facilitates distant organ metastasis [405].

#### Additional mechanisms

In addition, PRMTs facilitate tumor metastasis by modulating the expression, activity, and subcellular localization of transcription factors, thereby reprogramming the transcriptional landscape of oncogenes and tumor suppressors [406–408]. For instance, PRMT1 methylates HMG box protein 1 (HBP1) at R378, promoting its ubiquitination and degradation, thereby weakening its tumor-suppressive function and enhancing cancer cell proliferation and metastasis [406]. PRMT5 promotes gastric cancer progression by recruiting DNMT3A to the promoter region of the tumor suppressor gene IRX1, enhancing its DNA methylation and silencing its expression [407]. In breast cancer, PRMT6 catalyzes asymmetric dimethylation of STAT3 at R729, which enhances its membrane localization, interaction with JAK2, and phosphorylation at Y705, thereby promoting metastatic potential [408].

### Reprogramming energy metabolism

To adapt to hypoxia and nutrient deprivation, tumor cells undergo metabolic reprogramming to meet their energy, biosynthetic, and redox demands. This reprogramming is characterized by enhanced glycolysis, glutamine and lipid metabolism, activation of the pentose phosphate pathway (PPP), and the remodeling of the tricarboxylic acid cycle and amino acid metabolism [409, 410]. PRMTs contribute to these processes by directly methylating metabolic enzymes or regulating their expression, thereby reshaping tumor metabolism (Fig. 5; Table 1).

Glycolysis is a central metabolic pathway in cancer, and tumor cells often enhance glycolytic flux to meet their increased energy and biosynthetic demands for rapid proliferation [411]. Dysregulation of key enzymes—including GAPDH, enolase 1 (ENO1), pyruvate kinase M2 (PKM2), and lactate dehydrogenase A (LDHA)—drives tumor growth and metastasis [412, 413]. PRMTs regulate the expression and enzymatic activity of these factors via arginine methylation, thereby driving metabolic reprogramming and conferring growth advantages to tumor cells. The phosphofructokinase-fructose bisphosphatase (PFKFB) family controls levels of fructose 2,6-bisphosphate, which allosterically activates PFK-1 to enhance glycolytic throughput [414]. In HCC, PRMT1 methylates PFKFB3, promoting glycolysis and tumor progression [255]. GAPDH, a key glycolytic enzyme, is methylated by PRMT3 at R248 to enhance catalytic activity, whereas PRMT4-mediated methylation at R234 inhibits its function [415–417]. PGK1 catalyzes the first ATP-generating step of glycolysis. PRMT1 methylates PGK1 at R206, which promotes S203 phosphorylation, suppresses mitochondrial respiration, and shifts metabolism toward glycolysis [418, 419]. ENO1, which converts 2-phosphoglycerate to phosphoenolpyruvate, is methylated by PRMT6 at R9 and R372, enhancing dimerization and substrate affinity, thereby boosting enzymatic activity [420, 421]. PKM2, a rate-limiting enzyme in glycolysis and key regulator of tumor metabolism, is methylated by PRMT4, which inhibits calcium influx from the ER into mitochondria and promotes aerobic glycolysis over oxidative phosphorylation [422]. Additionally, PRMT6 methylates CRAF at R100, preventing its interaction with RAS and facilitating nuclear translocation of PKM2, which contributes to glycolytic reprogramming in HCC [423]. LDHA, the terminal enzyme in glycolysis that converts pyruvate to lactate, is activated by PRMT3-mediated methylation at R112, further promoting glycolytic metabolism in HCC cells [424]. Beyond direct enzymatic regulation, PRMTs also influence glycolysis indirectly by modulating key transcription factors (e.g., HIF-1α) [425] and oncogenic signaling pathways such as YAP, Myc, NF-κB, and ERK–PKM2 [423, 426–428].

The PPP is a glucose oxidation pathway that operates in parallel with glycolysis and primarily generates ribose-5-phosphate and NADPH—molecules critical for nucleotide biosynthesis and cellular redox homeostasis [429]. Under glucose limitation, the NRF2–PRMT4 axis activates H3R17me2, upregulating G6PD and redirecting glucose flux into the PPP [430]. In lung cancer, PRMT6 methylates 6PGD at R324, enhancing its enzymatic activity and increasing oxidative PPP flux [421]. In CRC, glucose deprivation induces PRMT4-dependent methylation of RPIA at R42, boosting catalytic efficiency and NADPH production, thereby supporting ROS clearance and nucleotide synthesis under nutrient stress [431].

Reprogramming of lipid metabolism is a hallmark of malignant tumors. Tumor cells support rapid growth by increasing lipid uptake and synthesis, with lipids also playing crucial roles in membrane structure, signal transduction, and energy supply [432]. Intracellular lipid metabolism and homeostasis are primarily regulated by sterol regulatory element-binding proteins (SREBPs) [433]. The PRMT5–MEP50 complex methylates SREBP1a, enhancing cholesterol, fatty acid, and triglyceride biosynthesis, thereby driving tumor growth and metastasis [275]. ATP citrate lyase (ACLY), a key enzyme involved in glucose metabolism, cholesterol and fatty acid synthesis, and inflammatory cascades, is also regulated by PRMT5. Specifically, PRMT5-mediated methylation of G3BP2 at R468 enhances its stability, which in turn stabilizes ACLY activation, promoting de novo lipid synthesis and contributing to the progression of HNSCC [434].

Amino acid metabolism reprogramming is intricately linked to protein and nucleotide synthesis, signaling pathway regulation, metabolic control, oxidative stress balance, and epigenetic modifications in tumor cells [435]. Glutamine serves as a major carbon and nitrogen donor for nucleotide, lipid, and protein synthesis, as well as for the production of other non-essential amino acids [436–438]. In PDAC, PRMT4 methylates the glutamine metabolism regulator MDH1 at R248, inhibiting its activity, reducing mitochondrial respiration and glutamine metabolism, thereby increasing cellular sensitivity to oxidative stress and suppressing proliferation [439]. Serine functions as the main source for glycine synthesis and is a critical donor of one-carbon units in tumor metabolism. Cancer cells sustain rapid proliferation by importing serine or synthesizing it de novo via PHGDH [440–442]. PRMT1 methylates PHGDH at R20, R54, and R236, modulating its enzymatic activity and regulating the serine biosynthesis pathway [286, 443]. Glycine, a precursor for glutathione, purines, creatine, and heme biosynthesis, is acquired from serum or generated through metabolism of serine, choline, or, in some mammals, threonine [444]. In chronic myeloid leukemia (CML), PRMT7 deletion downregulates glycine decarboxylase expression, disrupting glycine metabolism, leading to methylglyoxal accumulation and selectively eliminating CML stem cells without harming normal hematopoiesis [445].

### Evading immune destruction

Cancer cells can evade immune surveillance through multiple mechanisms, allowing persistent proliferation in the host [446]. These include reduced tumor immunogenicity, antigen mimicry, altered antigen expression, immune exclusion, and the establishment of an immunosuppressive microenvironment [447]. Genetic mutations and epigenetic reprogramming not only drive tumor progression but also underpin immune evasion [448]. PRMTs contribute to these processes by regulating innate immune signaling, and the expression of PD-L1 and MHC-I, ultimately influencing immune cell infiltration and anti-tumor immunity (Fig. 5; Table 1).

The cGAS–STING pathway is a pivotal cytosolic DNA-sensing mechanism that triggers type I interferon production and proinflammatory responses [449–451]. Cytosolic DNA, often derived from damaged DNA or ruptured micronuclei, is sensed by cGAS, which synthesizes cGAMP to activate STING and downstream TBK1–IRF3 and NF-κB signaling, inducing IFNs and cytokines [452]. PRMTs regulate this pathway at multiple levels: PRMT3 preserves mitochondrial DNA (mtDNA) integrity via HSP60 methylation, preventing mtDNA leakage [453]; PRMT9 modulates DNA repair, indirectly influencing cytosolic DNA accumulation [454, 455]; PRMT1 methylates cGAS at R133, disrupting its dimerization and downstream signaling [456]; and PRMT5 methylates IFI16, thereby suppressing DNA-induced IFN and chemokine production [457]. The RLR pathway senses cytosolic RNA and is crucial for antiviral and anti-tumor immunity [458]. PRMT1 represses endogenous retroviral elements (ERV)-derived dsRNA generation by epigenetically silencing DNMT1, thereby attenuating RLR activation [459]. PRMT1 inhibition combined with anti-PD-1 therapy enhances CD8⁺ T cell responses and suppresses melanoma growth. PRMT7 negatively regulates RLR signaling by repressing RIG-I, MDA5, and their dsRNA ligands, impairing antigen presentation and T cell activation [460]. BAF155 is a core subunit of the SWI/SNF chromatin remodeling complex, which regulates nucleosome positioning and chromatin accessibility in an ATP-dependent manner to control gene expression [92, 461]. In breast cancer, PRMT4 catalyzes the methylation of BAF155 (me-BAF155), leading to the repression of genes involved in the interferon-α/γ signaling pathway and attenuation of the host immune response [462]. Inhibition of PRMT4 or disruption of me-BAF155 enhances cytotoxic T cell function and tumor infiltration, thereby promoting anti-tumor immunity [462].

Immune checkpoint blockade (ICB) has revolutionized cancer therapy, demonstrating significant clinical efficacy across a wide spectrum of malignancies [463]. PD-L1, a key immune checkpoint molecule, is expressed on the surface of tumor cells and binds to PD-1 on T cells, leading to T cell dysfunction and immune evasion [452]. Although therapeutic blockade of the PD-1/PD-L1 axis leads to significant clinical improvement, the response rate is limited to approximately 20–40% of patients [464].Recent studies have identified pivotal roles for PRMT1 and PRMT5 in regulating PD-L1 expression [404, 465–467]. A promoter polymorphism (rs975484) in PRMT1 influences PD-L1/PD-L2 expression in HCC [465]. PRMT1 also stabilizes PGC-1α through methylation, which cooperates with STAT3 to transcriptionally activate PD-L1, promoting immune evasion in nasopharyngeal carcinoma [404]. PRMT5 regulates PD-L1 expression through symmetric dimethylation of H4R3 and H3R2, influencing gene expression either directly or indirectly. This epigenetic mechanism contributes to immune evasion in lung and cervical cancers [466, 467]. Furthermore, in castration-resistant prostate cancer, PRMT5 inhibition synergizes with anti-PD-1 therapy to enhance anti-tumor activity [468]. Resistance to ICB remains a major challenge, with impaired MHC-I antigen presentation representing one key mechanism [469]. In malignant melanoma, PRMT5 suppresses the transcription of NLRC5, a key transactivator of MHC-I genes, thereby downregulating the expression of genes involved in antigen processing and presentation and impairing tumor immunogenicity [457]. In prostate cancer, PRMT5 also epigenetically activates the expression of CSF2, promoting the infiltration of immunosuppressive neutrophils and monocytes. This contributes to the persistence of dysfunctional T cells and further dampens anti-tumor immune responses [470].

### Tumor-promoting inflammation

Chronic inflammation is a well-recognized driver of tumor initiation and progression. While inflammation originally evolved to eliminate pathogens and abnormal cells, persistent inflammatory signaling within the TME paradoxically fosters cancer development by activating transcription factors such as NF-κB, STAT3, and HIF-1α. These factors induce pro-inflammatory cytokines and mediators that promote tumor growth, invasion, and immune evasion [471–473]. Among them, NF-κB plays a central role by regulating genes involved in proliferation, angiogenesis, EMT, and immune modulation through both canonical and non-canonical pathways [474–478]. The NF-κB family members share a conserved Rel homology domain that mediates DNA binding and dimerization [477, 479]. Dysregulation of NF-κB signaling in cancer has been extensively linked to PTMs of the p65 subunit, with phosphorylation identified as a major regulatory mechanism [480]. More recently, arginine methylation of p65 has emerged as an important modulator of its DNA-binding affinity, nuclear localization, and interaction with co-activators [123, 124, 481–483]. In AML, PRMT2 functions as a key regulator of inflammation, and its loss leads to the activation of NF-κB- and STAT3-associated proinflammatory pathways [484]. In CRC, PRMT5 directly methylates p65 at R30 to enhance NF-κB transcriptional activity, while its knockdown attenuates this effect [124, 251]. In addition, PRMT5 also methylates YBX1 at R205, a modification required for YBX1-driven NF-κB activation and downstream gene expression [485].

### Unlocking phenotypic plasticity

Cellular plasticity is a fundamental process in embryonic development and is also evident in tissue repair and various diseases, enabling cells to remodel their phenotype in response to environmental changes [486]. In cancer, such plasticity is driven by microenvironmental cues, epigenetic modifications, and therapy-induced selective pressures, fueling tumor heterogeneity and resistance [383]. Increasing evidence indicates that cancer cells evade or reverse terminal differentiation by unlocking normally restricted plasticity, making this a key mechanism underlying tumor progression [280]. While EMT represents a well-studied form of plasticity discussed earlier, another clinically relevant manifestation is the acquisition of cancer stem cell (CSC)-like properties [383]. CSCs represent a subpopulation of tumor cells with self-renewal and multilineage differentiation potential, acting as central drivers of tumor progression and therapeutic resistance [487]. Phenotypic plasticity supports the maintenance of CSC stemness and facilitates the transition from non-CSCs to CSC-like states, thereby increasing tumor heterogeneity and resistance [488]. CSC biology is regulated by interconnected networks, including pluripotency transcription factors (OCT4, SOX2, Nanog, KLF4, Myc), intracellular signaling pathways (Wnt, NF-κB, Notch), epigenetic mechanisms, the tumor microenvironment, and EMT processes [489, 490]. Elucidating the molecular circuits that sustain CSC stemness, plasticity, and resistance is crucial for advancing CSC-targeted therapies [491].

Tumor cells can acquire or reinforce stem-like properties through reactivation of pluripotency-associated transcription factors such as OCT4, SOX2, Nanog, KLF4, and Myc [489, 490, 492]. PRMTs modulate CSC behavior by regulating the expression or activity of these factors. For instance, in small cell lung cancer, PRMT1 forms a complex with EphA2 to methylate SOX2, enhancing its transcriptional activity and promoting stemness [493]. In breast cancer, PRMT5 regulates OCT4/A, KLF4, FOXP1, and c-Myc expression to promote CSC proliferation, self-renewal, and resistance [494, 495]. Additionally, PRMT5 methylates and stabilizes KLF5, which enhances Nanog and OCT3/4 transcription, maintaining basal-like breast CSC function [496, 497]. PRMT8 also influences pluripotency factors like Nanog, OCT4, and SOX2, promoting colon CSC phenotypic transformation and tumor progression [498]. Multiple signaling pathways govern CSC maintenance and induction [489]. Among them, the Wnt pathway—comprising the canonical (β-catenin-dependent) and non-canonical (β-catenin-independent) branches—plays a central role [499]. Canonical Wnt signaling is essential for the maintenance of stem cells in tissues such as the intestine, breast, skin, and hair follicles [500, 501]. In CML, PRMT5 inhibition decreases leukemia stem cell survival and self-renewal by promoting DVL3 degradation and weakening Wnt/β-catenin signaling [502]. Conversely, PRMT5 deficiency in a gastric cancer mouse model increases Lgr5^+^ stem cells and activates Wnt/β-catenin, indicating tumor-type specific roles [503]. Other PRMTs also modulate Wnt signaling: PRMT2 promotes WNT5A expression [289]; PRMT1 activates β-catenin via MLXIP complex [256]; PRMT4 co-regulates β-catenin transcription with HIF-1α [295]; PRMT6 activates Wnt/β-catenin by inhibiting APC and GSK3β through the CDK9-YTHDF2 axis [177]. The RAF/MEK/ERK pathway is another critical regulator of CSC proliferation, self-renewal, and therapy resistance [504–506]. In HCC, PRMT6 methylates CRAF at R100, inhibiting its interaction with RAS and attenuating MEK/ERK signaling, thereby downregulating CD133, SOX2, and Nanog and reducing stemness [506]. Mitochondria, as hubs of metabolism, signaling, and cell survival, play pivotal roles in CSC persistence and therapeutic resistance [507, 508]. PRMT1 influences mitochondrial function and CSC traits via multiple pathways. In HCC, PRMT1 recruits TBX19 to the mitochondrial fission factor (MFF) promoter, inducing H4R3me2a and H3K9ac marks to activate MFF transcription, thereby enhancing mitochondrial fission and CSC self-renewal [509]. In breast cancer, PRMT1 methylates the RNA helicase DDX3, stabilizing the protein and facilitating its mitochondrial translocation, where, under stress conditions, DDX3 promotes PINK1 translation to drive CSC-like traits and metastatic progression [510].

### Genome instability and mutation

**Fig. 7:**
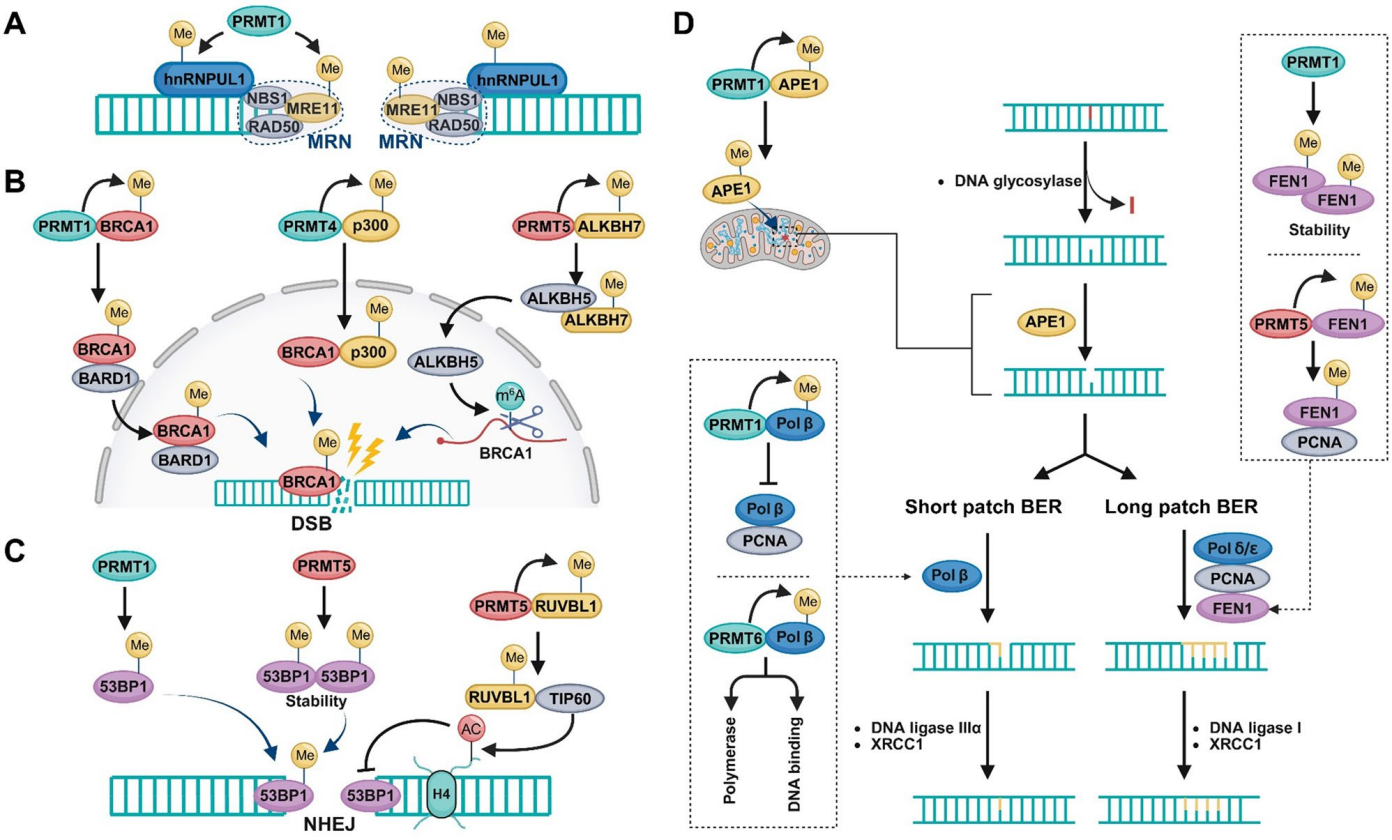
Roles of PRMTs in the DNA Damage Response. (**A**) PRMT1 promotes homologous recombination (HR) repair by methylating MRE11 and hnRNPUL1, facilitating DNA end resection and repair pathway activation. (**B**) PRMT1 directly methylates BRCA1, while PRMT4 enhances BRCA1 chromatin recruitment by methylating p300. Additionally, PRMT4-mediated methylation of ALKBH7 facilitates ALKBH5 nuclear translocation, which reduces m^6^A RNA methylation of BRCA1 mRNA, thereby enhancing its stability and expression. (**C**) PRMTs regulate non-homologous end joining (NHEJ) by controlling the localization, stability, and activity of 53BP1. Specifically, PRMT1 methylates 53BP1 to promote its recruitment to DNA damage sites and preserve its DNA-binding capacity. PRMT5 stabilizes 53BP1 through direct methylation and also methylates RUVBL1, activating TIP60-mediated H4K16 acetylation, which facilitates the release of 53BP1 from DSB sites. (**D**) PRMTs contribute to base excision repair (BER) by methylating key repair proteins. For example, PRMT1 methylates APE1, limiting its mitochondrial translocation and mitochondrial DNA repair function. PRMT1 also inhibits Pol β interaction with PCNA via methylation, while PRMT6 enhances Pol β DNA binding and polymerase activity. PRMT1 maintains FEN1 protein stability through methylation, and PRMT5 facilitates FEN1–PCNA interaction via methylation

DNA is vulnerable to damage from various endogenous and exogenous sources, including metabolic byproducts, environmental carcinogens, and chemical mutagens [511]. Such damage can impede genome replication and transcription, and if left unrepaired or improperly repaired, may result in mutations or extensive genomic alterations, posing a serious threat to cell and organismal survival [512]. In response, normal cells activate DNA damage checkpoints and engage multiple repair pathways—a coordinated process collectively termed the DDR [513]. Depending on the damage type and repair mechanism, DDR primarily involves direct reversal, base excision repair (BER), nucleotide excision repair, mismatch repair, and homologous recombination (HR) repair [514]. Deficiencies in DDR contribute to genomic instability, a major driver of tumorigenesis [279]. Genes encoding DDR factors are frequently mutated in various cancers, leading to impaired genomic integrity that both facilitates tumor growth and metastasis and increases sensitivity to DNA-damaging treatments such as radiotherapy [515]. PRMTs play a crucial role in preserving genomic stability and orchestrating the DDR through the methylation of key DDR regulators (Figs. 5 and 7; Table 1).

#### HR repair

DNA double-strand breaks (DSBs) represent severe cytotoxic lesions that, if not promptly or correctly repaired, can lead to mutagenic events or cell death [516]. Repair primarily occurs through non-homologous end joining (NHEJ), alternative end joining (Alt-EJ), or HR, the latter being the most faithful mechanism for restoring original DNA sequences [517, 518]. In mammals, DNA end resection is initiated by the MRE11/RAD50/NBS1 (MRN) complex and further facilitated by phosphorylation of CtBP-Interacting Protein (CtIP) [519]. Within this complex, MRE11 undergoes PRMT1-mediated arginine methylation, a modification essential for its exonuclease activity and S-phase checkpoint regulation, though not for complex assembly (Fig. 7A) [520]. hnRNPUL1, which binds the MRN complex and promotes BLM helicase recruitment, is similarly dependent on PRMT1-mediated methylation for its interaction with NBS1 and localization to DNA damage sites (Fig. 7A) [521, 522]. BRCA1, a key regulator of HR-mediated DSB repair, suppresses the error-prone NHEJ pathway by removing chromatin-associated 53BP1 and promoting DNA end resection [513, 523, 524]. Radiation therapy exerts its therapeutic effect primarily by inducing DNA damage in rapidly proliferating cancer cells [525]. Upon radiation exposure, PRMT1 methylates BRCA1, enhancing its interaction with BARD1 and promoting BRCA1 accumulation in the nucleus, thus regulating the DDR process (Fig. 7B) [525]. Additionally, PRMT4 methylates the histone acetyltransferase p300, facilitating BRCA1 recruitment to promoters of target genes, such as the cell cycle regulator p21, during DNA damage (Fig. 7B) [526]. PRMT5 regulates BRCA1 mRNA stability by promoting nuclear translocation of ALKBH5, which demethylates BRCA1 transcripts under doxorubicin treatment, thereby enhancing repair capacity and reducing chemotherapy efficacy in breast cancer cells (Fig. 7B) [527].

#### NHEJ repair

In NHEJ, DSBs are initially recognized by the Ku heterodimer, which subsequently recruits and activates the DNA-dependent protein kinase catalytic subunit (DNA-PKcs). This activation facilitates the assembly of terminal processing enzymes, DNA polymerases, and DNA ligase IV at the damage site to mediate end joining [512]. The protein 53BP1 restricts DNA end resection, generating overhangs suitable for ligation by the XRCC4–XLF–DNA ligase IV complex [512, 514, 515]. PRMT1 methylates 53BP1 at a motif essential for its recruitment and DNA-binding activity (Fig. 7C) [520]. PRMT5 similarly methylates and stabilizes 53BP1, supporting cell survival following DNA damage (Fig. 7C) [528]. Additionally, PRMT5-mediated methylation of RUVBL1 promotes TIP60 acetyltransferase activity, facilitating H4K16 acetylation and 53BP1 dissociation from DSBs (Fig. 7C) [106].

#### BER

The BER pathway addresses mild DNA damage by excising damaged bases, AP sites, and single-strand breaks (SSBs) [529]. The process is initiated by damage-specific DNA glycosylases, which hydrolyze N-glycosidic bonds to remove abnormal bases. Subsequently, AP endonuclease 1 (APE1) cleaves the DNA backbone, generating an SSB [514]. BER can be divided into two types based on the gap size: short-patch BER, primarily mediated by DNA polymerase β (Pol β), and long-patch BER, which involves Pol δ or Pol ε along with PCNA and FEN1 [514, 530, 531]. The gap is ultimately sealed by DNA ligase IIIα/XRCC1 complex in short-patch BER or DNA ligase I in long-patch BER [530, 532, 533]. Recent studies highlight the pivotal roles of PRMTs in regulating key BER factors. PRMT1 methylates APE1 at R301, facilitating its interaction with the outer mitochondrial membrane, which promotes APE1 translocation to mitochondria and enhances mitochondrial DNA repair capacity (Fig. 7D) [534]. Although current evidence for the involvement of PRMTs in regulating Pol β activity primarily stems from non-cancer contexts, these findings provide mechanistic insights that may be relevant to tumor-associated DNA repair processes. PRMT1-mediated methylation of Pol β may regulate its participation in PCNA-dependent DNA metabolic processes, influencing BER efficiency (Fig. 7D) [535]. PRMT6 methylates Pol β at R83 and R152, significantly increasing its DNA-binding affinity and polymerase elongation activity, thereby enhancing its polymerase function without notably affecting its single-nucleotide insertion or dRP lyase activities (Fig. 7D) [536]. Regarding FEN1 regulation, PRMT1-mediated methylation stabilizes FEN1 protein levels, improving DNA repair capacity and therapy resistance in lung cancer cells (Fig. 7D) [537]. Meanwhile, PRMT5 methylates FEN1, which inhibits phosphorylation at S187, strengthens the FEN1-PCNA interaction, and further promotes DNA replication and repair processes (Fig. 7D) [538].

### Non-mutational epigenetic reprogramming

In addition to genetic mutations, cancer cells undergo epigenetic reprogramming through non-mutational mechanisms, including DNA methylation, histone modifications, and alterations in chromatin accessibility [279, 280]. As pivotal epigenetic regulators, PRMTs play multifaceted roles in tumorigenesis and cancer progression by modulating gene expression through diverse mechanisms. PRMTs not only regulate chromatin architecture and gene transcription by catalyzing arginine methylation of histones, chromatin remodeling complexes, and transcription factors, but they also engage in cross-talk with other histone PTM and indirectly influence DNA methylation, thus participating broadly in epigenetic reprogramming. Moreover, PRMTs serve as key regulators across the mRNA lifecycle—including splicing, nuclear export, stability, and translation—through methylation of splicing factors, RBPs, and translation initiation factors.

### Senescent cells

Cellular senescence is a state of permanent cell cycle arrest that proliferating cells enter in response to various stressors, serving as a protective mechanism to prevent excessive damage accumulation [539]. Senescent cells are characterized by their loss of responsiveness to mitogenic stimuli, an enhanced secretory phenotype, and resistance to cell death [539]. As a stress response, senescence inhibits the proliferation of damaged or abnormal cells, playing crucial roles in embryonic development, tissue homeostasis, and cancer prevention [540]. In cancer, both senescent tumor cells and stromal cells can activate the p53/p21 and p16/Rb pathways, leading to irreversible proliferative arrest and halting the progression of precancerous lesions [541]. Senescent cells also secrete factors such as IL-6 and CXCL1 that recruit immune cells to eliminate potentially malignant cells [542]. However, the senescence-associated secretory phenotype (SASP) may paradoxically promote tumor growth, invasion, genomic instability, immune evasion, and drug resistance [540]. In breast cancer, PRMT1 promotes EMT by activating ZEB1 transcription via H4R3me2a modification, whereas PRMT1 silencing induces G1 arrest and cellular senescence [390]. In ovarian cancer, DNA-PK phosphorylates PRMT1 in response to cisplatin, recruiting it to pro-inflammatory gene promoters where it induces H4R3me2a and activates the senescence-associated secretory phenotype [240]. Meanwhile, PRMT6 binds to the p21 promoter and suppresses p21 expression via H3R2me2a methylation, thereby inhibiting cell cycle arrest and senescence in a p53-independent manner and promoting breast cancer cell proliferation [315]. Although these studies suggest that PRMTs modulate tumor-associated senescence, current research remains limited, highlighting the need for further investigation.

## Targeting PRMTs in tumor therapy

### PRMT inhibitors and research progress

With increasing insights into the physiological and pathological roles of PRMTs, these enzymes have emerged as promising targets for anticancer drug development [139, 268, 543]. Advances in structure-based drug design and high-throughput screening have enabled the discovery of small-molecule and peptide-based PRMT inhibitors with demonstrated antitumor activity [544]. Mechanistically, PRMT inhibitors are commonly classified as AdoMet-competitive, substrate-competitive, or allosteric agents [15, 545–547]. Beyond these conventional approaches, innovative strategies such as dual-target and covalent inhibitors, as well as protein degradation-based therapies, are gaining momentum. Among them, proteolysis-targeting chimeras (PROTACs) stand out by selectively degrading target proteins through the ubiquitin–proteasome system, offering enhanced potency and selectivity while mitigating toxicity and drug resistance [548–550]. These emerging technologies are expected to overcome the limitations of conventional PRMT inhibitors in terms of specificity, efficacy, and safety, thereby accelerating the clinical translation of PRMT-targeted therapies [551, 552].

#### Type I PRMTs

Type I PRMTs and their substrates have been implicated in the pathogenesis of various human cancers, underscoring their therapeutic potential [553]. Numerous inhibitors have been identified, though most remain at the preclinical stage, with only a few advancing into early clinical testing [15, 544]. These compounds can be broadly categorized into pan–Type I and selective inhibitors. Pan-Type I inhibitors, such as MS023, potently inhibit PRMT1, PRMT3, PRMT4, PRMT6, and PRMT8 at low nanomolar concentrations, with minimal activity against Type II and III PRMTs [554]. Another broad-spectrum inhibitor, AMI-1, displays micromolar-level activity against multiple PRMTs [555]. Dual-target inhibitors have also emerged; for example, AH237 selectively inhibits both PRMT4 and PRMT5 [556], while MS049 preferentially targets PRMT4 and PRMT6 [557]. In contrast to pan-inhibitors, selective inhibitors offer improved target specificity, potentially reducing off-target effects and toxicity, thereby enhancing therapeutic potential. GSK3368715 (also known as EPZ019997) is a selective PRMT1 inhibitor that demonstrated enhanced antitumor activity in MTAP-deleted cancer cells. Despite encouraging preclinical results, the phase I clinical trial (NCT03666988) of GSK3368715 was terminated early due to insufficient clinical efficacy and the emergence of thromboembolic events and dose-limiting toxicities [553, 558]. Another PRMT1 inhibitor, CTS2190, is currently undergoing a phase I/II trial to evaluate its safety and efficacy in patients with advanced or metastatic solid tumors (NCT06224387). Selective inhibitors targeting other Type I PRMTs are also under active development. PRMT4 inhibitors such as EZM2302 and TP-064 have shown promising antitumor activity in models of multiple myeloma, AML, and non-Hodgkin lymphoma [559–561]. Several PRMT6-selective inhibitors, including MS117, SGC-6870, EPZ020411, and the natural compound licochalcone A, have demonstrated therapeutic potential in various solid tumors, including glioblastoma, lung, gastric, and breast cancers [268, 562–564].

#### Type II PRMTs

##### PRMT5 inhibitors

Type II PRMT inhibitors, particularly those targeting PRMT5, have been extensively investigated in hematologic malignancies [565, 566]. Based on their developmental strategies and mechanisms of action, these inhibitors can be broadly classified into two generations. First-generation inhibitors primarily consist of non-selective agents, including both AdoMet-competitive inhibitors (such as LLY283, developed from nucleoside scaffolds) and substrate-competitive inhibitors (mainly derived from EPZ015666) [567, 568]. However, these compounds often suffer from poor selectivity, inhibiting other PRMTs and resulting in hematologic toxicity, which limits their clinical applicability. A representative example, GSK3326595, entered phase I clinical trials for breast cancer (NCT04676516), myelodysplastic syndromes and AML (NCT03614728), as well as solid tumors and non-Hodgkin lymphoma (NCT02783300), but all studies were terminated due to limited efficacy [569]. Another agent, JNJ-64,619,178, demonstrated potent anti-proliferative activity across various cancer models and durable PRMT5 inhibition in clinical settings; nevertheless, it yielded only modest benefits in patients with solid tumors, non-Hodgkin lymphoma, and low-risk MDS (NCT03573310) [570, 571]. Second-generation inhibitors exploit the metabolic vulnerability of MTAP-deficient cancers. MTAP loss causes accumulation of MTA, a natural PRMT5 inhibitor, which can be leveraged to achieve synthetic lethality [572–574]. For example, MRTX1719 has demonstrated potent antitumor activity in preclinical models of MTAP-deficient cancers and has shown objective responses in phase I/II trials for melanoma, gallbladder adenocarcinoma, mesothelioma, non-small cell lung cancer, and malignant peripheral nerve sheath tumors (NCT06672523, NCT05245500) [575, 576]. Another promising agent, AMG193, showed partial responses in 5 of 39 patients with advanced MTAP-deficient tumors, including esophageal, pancreatic, renal, gallbladder, and ovarian Sertoli-Leydig cell carcinomas (NCT06333951, NCT05094336, NCT06593522, NCT06360354) [577].

Novel approaches are expanding beyond traditional inhibition. PROTAC-based PRMT5 degraders, such as YZ-836P and MS4322, have been shown to selectively degrade PRMT5 and its downstream effectors, displaying robust antitumor effects [578, 579]. Additionally, dual-functional inhibitors targeting PRMT5 and EGFR or hnRNPE1 have shown promise. For instance, a PRMT5/EGFR dual inhibitor based on the 1-phenyl-tetrahydro-β-carboline scaffold exhibited potent antitumor activity in breast cancer models [580]. Another compound, 3 m, inhibited PRMT5 and upregulated hnRNPE1 expression, promoting apoptosis and suppressing cell migration and proliferation [581]. These innovative approaches aim to overcome the limitations of classical PRMT5 inhibitors by improving specificity and broadening therapeutic potential.

##### PRMT9 inhibitors

Currently, there are no selective inhibitors specifically targeting PRMT9. The most widely studied compound is MRK-990, a dual PRMT5/PRMT9 inhibitor developed by Merck in collaboration with the Structural Genomics Consortium (SGC) (source: SGC MRK-990).

#### Type III PRMTs

With growing insights into the biological functions of PRMT7 in various diseases, a series of highly selective small-molecule inhibitors have been developed. One of the most representative AdoMet-competitive PRMT7 inhibitors, SGC8158, exhibits potent inhibitory activity and high selectivity over other PRMTs [545]. Its prodrug, SGC3027, is efficiently converted to SGC8158 in vivo, enabling effective inhibition of PRMT7 under physiological conditions [460, 545]. Notably, SGC3027 has been shown to enhance antitumor immune responses and improve the efficacy of ICBs such as CTLA-4 and PD-1 in melanoma models. It also sensitizes tumor cells to bortezomib-induced apoptosis by blocking PRMT7-mediated monomethylation of HSP70 [460, 545]. Another promising compound, JS1310, targets PRMT7 by reprogramming glycine metabolism to elevate intracellular methylglyoxal levels, thereby impairing the self-renewal capacity of CML stem cells [445]. In addition, DS-437 is currently the first reported dual-target inhibitor with activity against both PRMT5 and PRMT7, demonstrating good selectivity and specificity in preclinical studies [582]. Despite these advances, PRMT7 inhibitors remain at the preclinical research stage, and none have yet entered human clinical trials. Further studies are required to comprehensively evaluate their pharmacokinetics, safety profiles, and therapeutic potential.

### Combination therapy

Combination therapy, which involves the concurrent use of multiple anticancer agents, is a cornerstone of modern cancer treatment [583]. By targeting multiple signaling pathways simultaneously, this approach can delay or overcome drug resistance, enhance therapeutic efficacy, and potentially reduce treatment-related toxicity [584]. Building upon the previously discussed roles of PRMTs and advances in PRMT inhibitor development, this section highlights the emerging potential of PRMT inhibition as a synergistic partner in chemotherapy, targeted therapy, and ICB.

#### Targeting PRMTs for sensitization to chemotherapy

Chemotherapy remains a fundamental strategy for eliminating rapidly proliferating tumor cells [585, 586]. However, its efficacy is frequently compromised by intrinsic or acquired resistance mechanisms [587, 588]. Accumulating evidence indicates that PRMTs contribute to chemotherapy resistance through diverse mechanisms. For instance, during cisplatin treatment, DNA-PK phosphorylates PRMT1 and recruits it to chromatin, where it promotes H4R3me2a deposition at pro-inflammatory gene promoters, thereby inducing SASP [240]. In gemcitabine-treated cells, PRMT1 fosters resistance both by disrupting the MAFF/BACH1 complex to drive epigenetic reprogramming and by methylating HSP70 to stabilize BCL2 expression [169, 589]. Similarly, PRMT3 methylates hnRNPA1, enhancing its binding to ABCG2 and stabilizing ABCG2 mRNA, ultimately promoting gemcitabine resistance [154]. These findings suggest that targeting PRMT1 or PRMT3 could help reverse resistance to cisplatin or gemcitabine. In addition, combining PRMT inhibitors with chemotherapeutic agents has shown synergistic effects. In lung cancer cells, PRMT5 inhibition with AMI-1 synergizes with cisplatin to reduce cell viability and induce apoptosis [590]. Furthermore, PRMT5 inhibitors are particularly effective in MTAP-deleted intrahepatic cholangiocarcinoma, where they enhance the efficacy of cisplatin and gemcitabine and suppress the growth of tumor organoids [591].

#### Targeting PRMTs for sensitization to targeted therapies

Targeted therapy, which selectively interferes with oncogenic drivers such as receptor tyrosine kinases, intracellular signaling proteins, or surface antigens, has transformed cancer management [592]. PRMT inhibitors have been shown to enhance tumor cell sensitivity to such agents and to act synergistically with multiple targeted drugs. For instance, inhibition of PRMT1 or treatment with PRMT1 inhibitors (e.g., AMI-1, C7280948) increases tumor sensitivity to the EGFR monoclonal antibody cetuximab and to PARP inhibitors such as olaparib [593, 594]. In glioblastoma and breast cancer models, PRMT5 inhibitors (e.g., EPZ015666, pemrametostat) have been shown to overcome resistance to mTOR inhibitors (e.g., PP242) and CDK4/6 inhibitors (e.g., abemaciclib, palbociclib, ribociclib) [113, 595]. Combination approaches also extend therapeutic indications. In MTAP-deficient non-small cell lung cancer, the type I PRMT inhibitor MS023 combined with the PARP inhibitor talazoparib induces greater DNA damage and significantly reduces cell viability [596]. In approximately 30% of AML patients, internal tandem duplication (ITD) mutations in the FLT3 gene are associated with poor prognosis [597]. Combining the PRMT1 inhibitor MS023 with the tyrosine kinase inhibitor AC220 markedly enhances the clearance of FLT3-ITD-positive AML cells [598]. Moreover, co-treatment with PRMT5 and PARP inhibitors restores antitumor activity in PARP inhibitor-resistant AML cells [599], while PRMT5 inhibition combined with AKT inhibitors synergistically suppresses the growth of diffuse large B-cell lymphoma [600].

#### Targeting PRMTs for sensitization to immunotherapy

ICB has emerged as a promising strategy for treating malignancies such as melanoma and non-small cell lung cancer. However, due to primary or acquired resistance, most patients fail to respond or achieve sustained clinical benefit [601–603]. A CRISPR-based screening coupled with multi-omics analysis identified PRMT1 as a key immune resistance factor that regulates both T cell infiltration and cytotoxic activity [604]. Targeting PRMT1 enhances tumor sensitivity to T cell-mediated killing and improves responses to anti-PD-1/OX40 therapy, highlighting its potential as a target to overcome ICB resistance. In preclinical models of PDAC and melanoma, PRMT1 inhibitors combined with anti-PD-1 or anti-PD-L1 antibodies increased CD8⁺ T cell infiltration, suppressed tumor growth, and partially reversed resistance to ICB therapy [459, 605, 606]. Other PRMTs also play immunomodulatory roles: in melanoma, the PRMT4 inhibitor EZF2302 restores sensitivity to anti-CTLA-4 therapy [607], while the PRMT7 inhibitor SGC3027 enhances anti-tumor T cell responses and reduces tumor burden when combined with ICB by promoting immune cell infiltration [460]. Collectively, these findings underscore the importance of PRMTs in shaping anti-tumor immunity and suggest that co-targeting PRMTs, especially PRMT1, may offer a promising approach to improve the efficacy of immune checkpoint therapies.

## Conclusions and future perspectives

PRMTs constitute a major family of enzymes that catalyze the methylation of arginine residues on histone and non-histone proteins, thereby regulating diverse biological processes, including embryonic development, tissue homeostasis, immune responses, and metabolism [608]. Functionally, PRMTs act across multiple molecular layers—such as transcription, RNA splicing, translation, and post-translational modifications—reflecting their central role in coordinating gene expression and cellular homeostasis [609, 610]. Moreover, their expression and activity are orchestrated by multifaceted mechanisms—ranging from transcriptional and post-transcriptional regulation to metabolic signaling—highlighting their central and precisely regulated role within the epigenetic network.

In the context of tumorigenesis and progression, PRMTs are implicated in virtually all hallmarks of cancer, including sustained proliferative signaling, evasion of growth suppressors, resistance to cell death, induction of angiogenesis, activation of invasion and metastasis, metabolic reprogramming, immune evasion, tumor-promoting inflammation, phenotypic plasticity, genome instability, epigenetic reprogramming, and senescence regulation. However, due to the diversity of PRMT substrates, tumor heterogeneity, and the variability of experimental systems and approaches, the precise mechanisms by which PRMTs function in different cancer types remain incompletely understood. Current studies are largely focused on expression profiles, with comparatively limited attention to the dynamic regulation of enzymatic activity and non-catalytic roles of PRMTs [191–194]. Future research should systematically investigate both the enzymatic and non-enzymatic functions of PRMTs to fully elucidate their multilayered roles in cancer development and progression. Despite ongoing debate, a growing body of foundational research has established a strong theoretical basis and promising therapeutic potential for targeting PRMTs in cancer treatment [611].

Drug resistance remains a major challenge in cancer treatment, necessitating the development of more effective strategies. Recent studies show that PRMTs promote tumor survival under therapeutic stress by regulating drug transport, DNA repair, stemness, metabolism, autophagy, and microenvironment remodeling, thereby enhancing immune evasion and chemotherapy resistance and representing promising targets to overcome treatment resistance [14]. The development of selective PRMT inhibitors offers new avenues to overcome resistance, but challenges remain [12, 14, 612]. For example, pan-PRMT inhibitors often suffer from off-target effects and lack specificity, limiting their clinical use, while AdoMet-competitive inhibitors face safety concerns and narrow therapeutic windows [12, 14, 15, 546]. High sequence homology and functional overlap among PRMT family members further complicate inhibitor selectivity [613, 614]. Therefore, precisely defining the roles of individual PRMT, especially regarding resistance-related epigenetic regulation, is critical for designing highly selective, low-toxicity inhibitors. Currently, several PRMT inhibitors have entered clinical development, with PRMT5 inhibitors being the most extensively investigated [552, 615, 616]. However, first-generation PRMT5 inhibitors have shown limited clinical efficacy and are frequently associated with hematologic toxicities—such as thrombocytopenia, anemia, and neutropenia—resulting in the early termination of some clinical trials [569–571, 617]. In contrast, second-generation PRMT5 inhibitors, including MTA-cooperative compounds and targeted protein degraders, are emerging as promising alternatives due to their enhanced selectivity and reduced toxicity profiles [575–579].Meanwhile, combining PRMT inhibitors with chemotherapy, targeted therapies, or immunotherapy has shown promise in enhancing treatment sensitivity and delaying resistance onset, representing a highly promising anti-resistance approach [590, 591, 596, 598, 600, 604].

Despite these advances, several critical questions remain. How do PRMTs selectively recognize and methylate specific substrates in distinct TMEs? How do PRMT-mediated methylation events interact with other epigenetic modifications, such as acetylation and phosphorylation, to coordinately regulate gene expression and cellular phenotypes? Addressing these key questions is crucial for a comprehensive understanding of the roles of PRMTs in tumor initiation, progression, and resistance. From a translational perspective, most current PRMT inhibitors face limitations such as poor selectivity, uncertain therapeutic efficacy, and notable side effects, which hinder their clinical application. Although several PRMT inhibitors have entered preclinical development, the majority remain at the in vitro validation stage, with insufficient studies on pharmacodynamics and pharmacokinetics—especially regarding efficacy and safety in animal models. Furthermore, mechanistic insights are largely derived from traditional animal models such as mice or rats, which often fail to recapitulate the biological complexity of human tumors. Therefore, the clinical translatability of these findings requires further validation. To bridge this gap, more clinically relevant platforms—such as patient-derived organoids and xenograft models—are urgently needed to better mimic TMEs and therapeutic responses, thereby enhancing the feasibility and application prospects of PRMT-targeted therapies.

With the rapid advancement of single-cell sequencing, spatial omics, and CRISPR-based functional screening, future studies are expected to reveal the spatial heterogeneity and regulatory networks of PRMTs across tumor cell subpopulations and microenvironmental niches. These technologies will facilitate the identification of predictive biomarkers and accelerate the clinical translation of PRMT-based precision medicine. Additionally, integrating structural biology with AI-assisted drug design may enable the development of highly selective, low-toxicity next-generation PRMT inhibitors. Systematic in vivo evaluations of their efficacy and safety will be essential for clinical validation. Given the intricate nature of epigenetic regulation, personalized therapeutic strategies based on PRMT expression profiles and substrate methylation patterns—combined with chemotherapy, targeted therapies, or immunotherapies—may help overcome drug resistance and improve outcomes in refractory or relapsed cancers. Overall, PRMTs are central players in the epigenetic regulatory network and hold both fundamental biological significance and translational promise. Continued systematic research will not only deepen our understanding of cancer epigenetics but also open new avenues for precise and individualized cancer therapies.

## Data Availability

No datasets were generated or analysed during the current study.

## Abbreviations

3’ UTR: 3’ untranslated region
53BP1: p53-binding protein 1
ACLY: ATP citrate lyase
ADMA: Asymmetric ω-N^G^,N^G^-dimethylarginine
ALT-EJ: Alternative end-joining
AML: Acute myeloid leukemia
ANGPT: Angiopoietin
APE1: AP endonuclease 1
AR: Androgen receptor
ARE: AU-rich elements
BAF155: BRM-associated factor 155
BER: Base-excision repair
BRG1: BRM-related gene 1
BRM: Brahma
CHD: Chromodomain helicase DNA-binding
CML: Chronic myeloid leukemia
CFIm: Mammalian cleavage factor I
CSC: Cancer stem cell
CtIP: CtBP-interacting protein
CTD: Carboxy-terminal domain
DDX3: DEAD-box polypeptide 3, X-linked
DDR: DNA damage response
DNA-PK: DNA-dependent protein kinase
DSBs: DNA double-strand breaks
ECM: Extracellular matrix
EMT: Epithelial-mesenchymal transition
ERV: Endogenous retroviral elements
FGFR3: fibroblast growth factor receptor 3
FLT3: FMS-like tyrosine kinase 3
FUS: Fused in sarcoma
H3K27me3: Histone H3 at lysine 27
H3K4me3: Trimethylation of histone H3K4
H3R2me1: Monomethylation of histone H3R2
H4R3me2a: Asymmetric dimethylation of histone H4R3
H4R3me2s: Symmetric dimethylation of H4R3
HBP1: HMG box protein 1
HCC: Hepatocellular carcinoma
HNSCC: Head and neck squamous cell carcinoma
hnRNP: Heterogeneous nuclear ribonucleoprotein
HR: Homologous recombination
ICB: Immune checkpoint blockade
INO80: Inositol 80
IRAlus: Inverted Alu repeats
ITD: Internal tandem duplication
LSD1: Lysine-specific histone demethylase 1
MAD2L2: Mitotic arrest deficient 2 like 2
MEP50: Methylosome protein 50
MFF: Mitochondrial fission factor
MMA: ω-N^G^-monomethylarginine
MMPs: Matrix metalloproteinases
m^6^A: N(6)-methyladenosine
mRNP: Messenger ribonucleoprotein particle
MTAP: Methylthioadenosine phosphorylase
MTA: Methylthioadenosine
NADPH: Nicotinamide adenine dinucleotide phosphate
ncRNAs: Non-coding RNAs
NHEJ: Non-homologous end-joining
NONO: Non-POU domain containing octamer binding
NuRD: Nucleosome remodeling and deacetylase
PDAC: Pancreatic ductal adenocarcinoma
PCD: Programmed cell death
PER3: Period circadian regulator 3
PFKFBs: Phosphofructokinase-fructose bisphosphatases
PHGDH: Phosphoglycerate dehydrogenase
PKM2: Pyruvate kinase M2
Pol II: RNA polymerase II
PPP: Pentose phosphate pathway
PRC2: Polycomb repressive complex 2
PRMT1: Protein arginine methyltransferase 1
PROTAC: Proteolysis targeting chimeras
PTMs: Post-translational modifications
RBPs: RNA-binding proteins
RPS2/3: Ribosomal protein S2/3
RUVBL1: RuvB-like protein 1
AdoMet: S-adenosylmethionine
AdoHcy: S-adenosylhomocysteine
SASP: Senescence-associated secretory phenotype
SDMA: Symmetric ω-N^G^,N^G^-dimethylarginine
SGs: Stress granules
SH3: Src homology 3
SMN: Survival motor neuron
snRNA: Small nuclear RNA
snRNPs: Small nuclear ribonucleoproteins
SREBPs: Sterol regulatory element-binding proteins
SWI/SNF: Switch/sucrose non-fermentable
TAD: Transcriptional activation domain
TIP60: Tat-interactive protein, 60 kDa
TME: Tumor microenvironment
TNBC: Triple-negative breast cancer
TR3: Testicular receptor 3
TSP-1: Thrombospondin 1
WDR5: WD repeat domain 5
